# Optimisation of ^1^H PMLG homonuclear decoupling at 60 kHz MAS to enable ^15^N–^1^H through-bond heteronuclear correlation solid-state NMR spectroscopy†‡

**DOI:** 10.1039/d2cp01041k

**Published:** 2022-07-26

**Authors:** Jacqueline Tognetti, W. Trent Franks, Józef R. Lewandowski, Steven P. Brown

**Affiliations:** Department of Chemistry, University of Warwick Coventry CV4 7AL UK; Department of Physics, University of Warwick Coventry CV4 7AL UK S.P.Brown@warwick.ac.uk

## Abstract

The Lee–Goldburg condition for homonuclear decoupling in ^1^H magic-angle spinning (MAS) solid-state NMR sets the angle *θ*, corresponding to arctan of the ratio of the rf nutation frequency, *ν*_1_, to the rf offset, to be the magic angle, *θ*_m_, equal to tan^−1^(√2) = 54.7°. At 60 kHz MAS, we report enhanced decoupling compared to MAS alone in a ^1^H spectrum of ^15^N-glycine with 
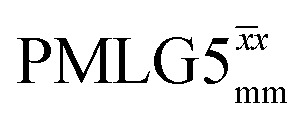
 at *θ* = 30° for a *ν*_1_ of ∼100 kHz at a ^1^H Larmor frequency, *ν*_0_, of 500 MHz and 1 GHz, corresponding to a high chemical shift scaling factor (*λ*_CS_) of 0.82. At 1 GHz, we also demonstrate enhanced decoupling compared to 60 kHz MAS alone for a lower *ν*_1_ of 51 kHz, *i.e.*, a case where the nutation frequency is less than the MAS frequency, with *θ* = 18°, *λ*_CS_ = 0.92. The ratio of the rotor period to the decoupling cycle time, *Ψ* = *τ*_r_*/τ*_c_, is in the range 0.53 to 0.61. Windowed 
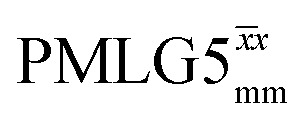
 decoupling using the optimised parameters for a *ν*_1_ of ∼100 kHz also gives good performance in a ^1^H spin-echo experiment, enabling implementation in a ^1^H-detected ^15^N–^1^H cross polarisation (CP)-refocused INEPT heteronuclear correlation NMR experiment. Specifically, initial ^15^N transverse magnetisation as generated by ^1^H–^15^N CP is transferred back to ^1^H using a refocused INEPT pulse sequence employing windowed 
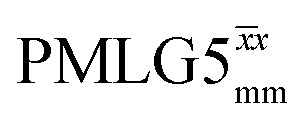
^1^H decoupling. Such an approach ensures the observation of through-bond N–H connectivities. For ^15^N-glycine, while the CP-refocused INEPT experiment has a lower sensitivity (∼50%) as compared to a double CP experiment (with a 200 μs ^15^N to ^1^H CP contact time), there is selectivity for the directly bonded NH_3_^+^ moiety, while intensity is observed for the CH_2_^1^H resonances in the double CP experiment. Two-dimensional ^15^N–^1^H correlation MAS NMR spectra are presented for the dipeptide β-AspAla and the pharmaceutical cimetidine at 60 kHz MAS, both at natural isotopic abundance. For the dipeptide β-AspAla, different build-up dependence on the first spin-echo duration is observed for the NH and NH_3_^+^ moieties demonstrating that the experiment could be used to distinguish resonances for different NH_*x*_ groups.

## Abstract 2

^15^N–^1^H heteronuclear NMR correlation at natural abundance in the solid state *via J* couplings is enabled by optimisation of phase-modulated Lee–Goldburg (PMLG) ^1^H homonuclear decoupling during the spin echoes, far from the ideal magic-angle condition.

## Graphical abstract

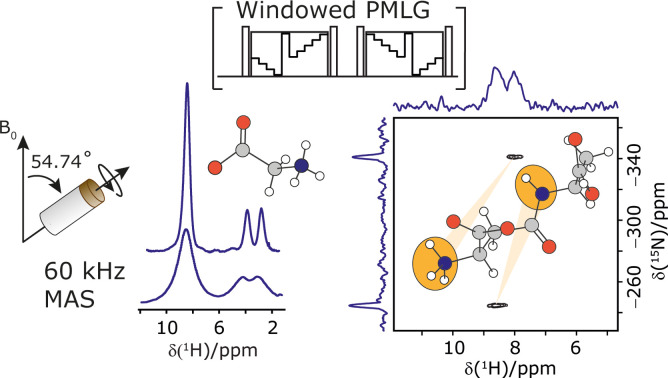

## 1. Introduction

Direct ^1^H detection is increasingly important for solid-state NMR study of pharmaceuticals^1–4^ and biological molecules.^5–8^ The availability of ever faster Magic Angle Spinning (MAS) frequencies reduces line broadening due to ^1^H homonuclear dipolar couplings.^9–14^ In particular, ^1^H detection is advantageous for the identification of specific correlations to nuclei with low gyromagnetic ratio, *γ*, such as the two natural-abundant isotopes of nitrogen, ^14^N and ^15^N. Our focus here is on the spin *I* = 1/2 ^15^N, though it is to be noted that there is increasing application of ^14^N–^1^H experiments for the much higher natural abundance (99.6%) spin *I* = 1 nucleus.^15–22^ The low sensitivity of ^15^N, associated with its low natural abundance and gyromagnetic ratio, can be overcome by the use of ^15^N–^1^H correlation experiments with proton acquisition, thanks to the high natural abundance and *γ* that characterise protons, provided that fast MAS can achieve sufficient ^1^H line narrowing.^23–26^ We note that an ^15^N-detected MAS-*J*-HMQC ^1^H–^15^N two-dimensional spectrum has also been recorded at natural abundance and 12.5 kHz MAS using Frequency Switched Lee–Goldburg (FSLG) ^1^H homonuclear decoupling.^27^^1^H-detected heteronuclear ^15^N–^1^H correlation experiments can be achieved by inverse polarization, CP, as applied to small molecules^23,25,26,28–30^ and ^15^N-labelled proteins as a hNH experiment.^31–33^ An alternative to CP-based dipolar-mediated through-space transfer is a *J* coupling mediated through-bond refocused INEPT solid-state NMR experiment.^34–37^ Specifically, we consider the CP-refocused INEPT correlation experiment,^38,39^ whereby *J* coupling mediated ^15^N–^1^H back-transfer, following CP to give maximum initial ^15^N magnetisation, ensures only the observation of peaks due to through-bond transfer in a ^15^N–^1^H spectrum.^26^ However, fast dephasing due to strong ^1^H homonuclear dipolar couplings shortens ^1^H coherence lifetimes, reducing sensitivity, making *J* coupling based experiments challenging. Even 60 kHz MAS is not sufficient to completely average out ^1^H homonuclear dipolar couplings.^40^ The application of ^1^H homonuclear decoupling^41–44^ under fast MAS during the ^15^N–^1^H coherence transfer improves sensitivity sufficiently for refocused INEPT transfer.^26,39^

While a large number of ^1^H homonuclear decoupling schemes have been optimised under static conditions for operation at low (5–10 kHz) and moderate (∼15 kHz) MAS frequencies;^41–54^ there have only been a few papers presenting ^1^H homonuclear decoupling at faster MAS frequencies of (35+ kHz)^55,56^ and (60+ kHz).^57–62^^1^H homonuclear decoupling is clearly not being applied under quasi-static conditions under such fast MAS and the performance is dependent upon the ratio between the rotor period, *τ*_r_, and the cycle time of the ^1^H homonuclear decoupling, *τ*_c_. Lee–Goldburg^45,46,49,59^ and DUMBO^50,62^ based decoupling are characterized by short cycle times which makes them compatible with faster MAS implementations. Nevertheless, a short cycle time means high ^1^H nutation frequencies, *ν*_1_, for the scheme which can be demanding on the instrumentation. In this work, we consider the application of phase-modulated Lee–Goldburg (PMLG)^49^ in a 1D ^1^H Combined Rotation and Multiple-Pulse Sequence (CRAMPS)^63^ experiment at 60 kHz MAS using relatively low nutation frequencies. The performance of PMLG depends on multiple factors such as the type of PMLG-block, frequency offset, and ^1^H nutation frequency;^41,42,53,54^^1^H homonuclear decoupling sequences are usually evaluated through three principal parameters: the chemical shift scaling factor (*λ*_CS_),^57,58,64^ and linewidth improvement reflected in sensitivity and resolution determined through observation of the chemical shift evolution,^62^ and extended coherence lifetimes as observed through spin-echo experiments.^57^ A bimodal Floquet theory analysis shows that ^1^H homonuclear decoupling requires a fine optimization at MAS above 40 kHz owing to the considerable number of zero- and first-order degeneracies.^65^ The two types of degeneracy arise when *nν*_r_ + *kν*_c_ = 0, where *ν*_r_ is the MAS spinning frequency and *ν*_c_ is the cycle frequency of the decoupling block, and *n* and *k* are integers. When these conditions are met, degeneracies occur within the diagonal block of the Floquet Hamiltonian and the effective Hamiltonian^66^ leading to dipolar line-broadening.

In this paper, we first demonstrate, at 60 kHz MAS, enhanced decoupling compared to MAS alone in a ^1^H solid-state NMR spectrum of ^15^N-glycine for an angle *θ*, corresponding to arctan of the ratio of the rf nutation frequency, *ν*_1_, to the rf offset, that is far from the ideal magic angle, *θ*_m_, equal to tan^−1^(√2) = 54.7°. Moreover, the application of windowed 
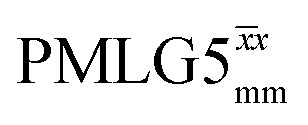
 decoupling with parameters based on those optimised for the one-pulse spectrum gives enhanced dephasing times in a ^1^H spin-echo experiment. In this way, we systematically investigate the ^1^H homonuclear decoupling parameters that affect sensitivity in the ^15^N–^1^H CP-refocused INEPT experiment under ^1^H homonuclear decoupling and fast MAS. It is shown that optimized decoupling enables the recording of two-dimensional through-bond ^15^N–^1^H MAS NMR correlation spectra for moderately sized organic molecules such as the dipeptide β-AspAla and the pharmaceutical cimetidine.

## 2. Experimental

^15^N-Labelled glycine, and natural abundance (NA) glycine, β-AspAla and cimetidine were purchased from Sigma Aldrich or Bachem (β-AspAla) and packed as received into 1.3 mm zirconia rotors. ^15^N-Glycine was packed into a restricted volume in the centre of the rotor using silicone spacers. ^15^N-Labelled glycine was used to optimise ^1^H homonuclear decoupling in 1D and 2D correlation experiments and the 2D ^15^N–^1^H CP-refocused INEPT experiment. Glycine, β-AspAla and cimetidine, all at natural abundance, were used to test the ^15^N–^1^H natural abundance CP-refocused INEPT correlation experiment.

The experiments were performed on a Bruker Avance III (500 MHz) or Avance NEO (600 MHz, 1 GHz) spectrometer operating at a ^1^H Larmor frequency of *ν*_0H_ = 500.13 MHz (11.7 T), 599.45 MHz (14.1 T), 1000.40 MHz (23.5 T) and sample spinning using a Bruker 1.3 mm HXY probe at 60 kHz. The 90° pulse duration of 2.5 μs (*ν*_1_ = 100 kHz) for ^1^H and 4 μs (*ν*_1_ = 62.5 kHz) or 3.5 μs (*ν*_1_ = 71.4 kHz, cimetidine) for ^15^N was calibrated using a one-pulse experiment and a CP followed by a 90° pulse experiment, respectively. A recycle delay of 3 s or 5 s (cimetidine) was used.

^1^H chemical shifts are externally referenced with respect to tetramethylsilane (TMS) *via*l-alanine at natural abundance as a secondary reference (1.1 ppm for the CH_3_^1^H resonance) corresponding to adamantane at 1.85 ppm.^67,68^^15^N chemical shifts are referenced relative to liquid CH_3_NO_2_ at 0 ppm,^69^ using the NH_3_^+^ peak of glycine (at natural abundance) at −347.4 ppm as a secondary reference. To convert to the chemical shift scale frequently used in protein NMR, where the alternative IUPAC reference (see Appendix 1 of ref. 70) is liquid ammonia at −50 °C, it is necessary to add 379.5 to the given values.^71^^1^H and ^15^N chemical shifts can be experimentally determined to an accuracy of ±0.2 and ±0.1 ppm, respectively. The ^15^N RF transmitter frequency was centred at −304.5 ppm (or −291.5 ppm cimetidine). Where the ^1^H resonance offset is referred to, 0 kHz refers to on-resonance with the NH_3_^+^ peak of glycine at 8.4 ppm, with a positive resonance offset referring to a move of the RF transmitter frequency to higher ppm.

### 1D CRAMPS

The acquisition window was optimized to acquire 40 complex data points, each corresponding to 0.1 μs, with a ringdown delay of 1.0 μs and a deadtime optimized to be 2.2 μs, corresponding to a total acquisition window, *τ*_w_, of 7.2 μs. The total acquisition time was 15 ms. Both 
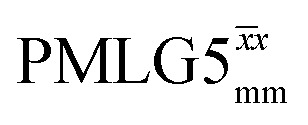
 and 
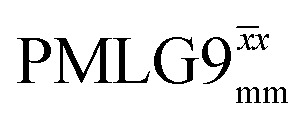
^1^H homonuclear decoupling schemes were optimized over a ^1^H nutation frequency, *ν*_1_(^1^H), range from ∼10 to ∼120 kHz.

### 2D ^15^N–^1^H CP-refocused INEPT

**Fig. 1:**
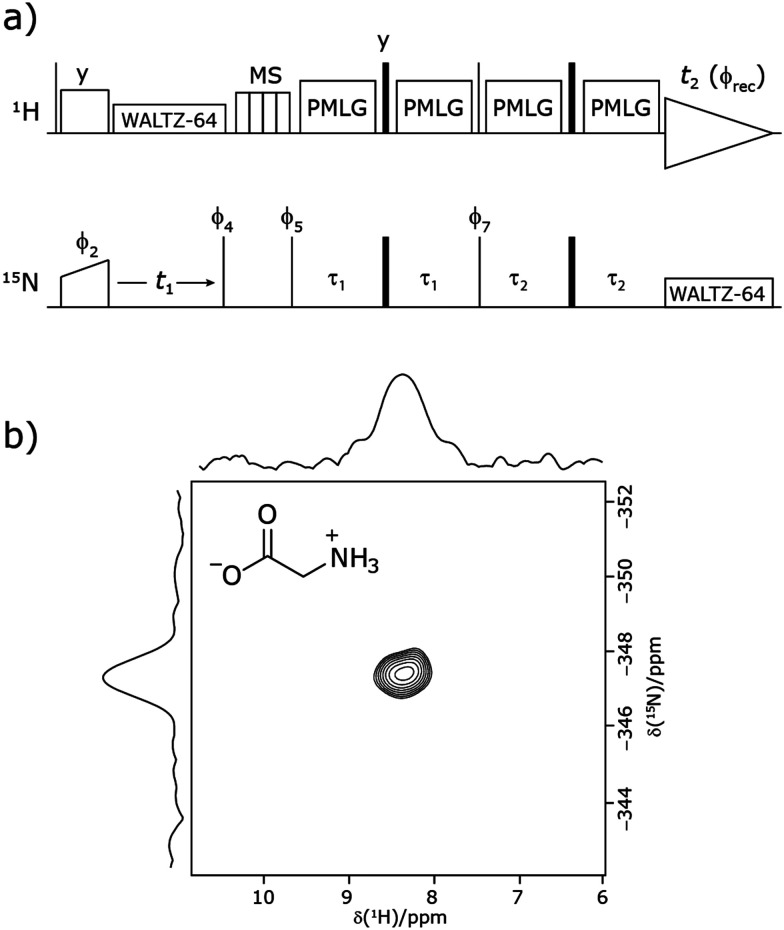
(a) Pulse sequence for the ^15^N–^1^H CP-refocused INEPT experiment utilised in this paper. Narrow lines and filled black rectangles represent π/2 and π pulses, respectively. Where not stated, the phase of a pulse is *x*. The following phase cycle is applied: *ϕ*_2_ = {x*2, −x*2}, *ϕ*_4_ = {−y*4, y*4}, *ϕ*_5_ = {y*8, −y*8}, *ϕ*_7_ = {*x*, −*x*} and acquisition *ϕ*_rec_ = {*x*, −*x*, −*x*, *x*, −*x*, *x*, *x*, −*x*, −*x*, *x*, *x*, −*x*, *x*, −*x*, −*x*, *x*}. States-TPPI is implemented on *ϕ*_4_. (b) A ^15^N–^1^H (*ν*_0_ = 500 MHz) 2D CP (contact time = 2 ms)-refocused INEPT MAS (*ν*_r_ = 60 kHz) NMR correlation spectrum with skyline projections of natural abundance glycine and its molecular structure. 
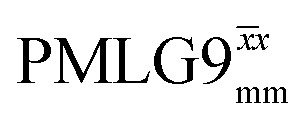
 was applied at a ^1^H nutation frequency of 106 kHz (*τ*_LG_ = 2.92 μs) during both *τ*_1_ = 2.091 ms (179*τ*_c_) and *τ*_2_ = 0.993 ms (85*τ*_c_) at a ^1^H transmitter offset of −2.6 kHz, with a zero offset corresponding to being on resonance with the NH_3_^+^ peak. 192 transients were coadded for each of 96 *t*_1_ FIDs, corresponding to a total experimental time of 16 hours. The base contour is at 40% of the maximum intensity.

Cross polarization (CP) from ^1^H to ^15^N was used for the initial excitation of ^15^N transverse magnetisation, where the ^1^H nutation frequency was ∼80 kHz (or ∼95 kHz for cimetidine) using a zero-quantum (ZQ) match condition;^72,73^ and a ^15^N nutation frequency of ∼20 kHz (or ∼25 kHz for cimetidine) with a linear ramp^74^ (70–100%) on the ^15^N channel (glycine and β-AspAla) or ^1^H (cimetidine). A CP contact time of 2 ms (or 4 ms for cimetidine) was used. The MISSISSIPPI suppression scheme^75^ was applied with a spinlock nutation frequency of ∼30 kHz for four intervals of 2 ms (or 5 ms for cimetidine) to remove residual ^1^H transverse magnetisation. Low-power^76^ heteronuclear ^1^H and ^15^N decoupling was applied during *t*_1_ evolution and ^1^H acquisition, respectively, using WALTZ64^77,78^ at a nutation frequency of ∼10 kHz. The pulse sequence used corresponds to a modified version of that presented by Althaus *et al.* (Fig. 1b).^26^

Each ^1^H-detected FID was acquired for 30 ms with a spectral width of 80 ppm (or 40 ppm for cimetidine). The ^15^N dimension was acquired with 96 (glycine NA and β-AspAla) or 64 (cimetidine) *t*_1_ FIDs with a dwell time of 300 μs (glycine NA) or 142 μs (β-AspAla) or 160 μs (cimetidine), corresponding to a ^15^N spectral width of 66 ppm (glycine NA) or 138 ppm (β-AspAla) or 102 ppm (cimetidine) and a maximum *t*_1_ of 15 ms (glycine NA), 6.9 ms (β-AspAla), or 5.1 ms (cimetidine). The States-TPPI method was employed to achieve sign discrimination in the indirect dimension.

## 3. Results and discussion

### 3.1 ^15^N–^1^H CP-refocused INEPT – pulse sequence and product operator analysis

Our implementation of the ^15^N–^1^H CP-refocused INEPT experiment at 60 kHz MAS is shown in Fig. 1a. Note that the pulse sequence in Fig. 1a corresponds to a modified version of that used by Althaus *et al.* at *ν*_r_ = 40 kHz.^26^ The pulse sequence begins with an initial ^1^H to ^15^N CP transfer to provide the largest pool of polarization possible for the low-γ and natural abundance ^15^N nucleus. The ^15^N transverse magnetisation is allowed to evolve during *t*_1_. The desired magnetisation is stored during a *z*-filter period, in which ^1^H magnetisation suppression using the MISSISSIPPI sequence^75^ is implemented to remove the background proton signals. A ^15^N–^1^H refocused INEPT element is used to transfer the magnetization back to proton for acquisition. INEPT utilizes the ^1^H–^15^N *J* couplings to restrict the signals observed to those with direct one-bond H–N connections. Each spin-echo duration should be an integer number of rotor periods to ensure that the chemical shift anisotropy is completely averaged by MAS. Homonuclear ^1^H decoupling, here PMLG,^49^ is applied during the two spin-echoes of the refocused INEPT element. Under fast MAS, at a spinning frequency of 60 kHz in this work, low power heteronuclear decoupling,^76^ specifically WALTZ-64^78^ decoupling, is applied on ^1^H and ^15^N during *t*_1_ and *t*_2_, respectively. The resulting spectrum is a 2D ^15^N–^1^H through-bond correlation spectrum, as illustrated in Fig. 1b for natural abundance glycine.

For a ^15^N–^1^H spin pair, a product-operator analysis (see Section S1,ESI‡) shows a product of sine terms dependence on the heteronuclear ^15^N–^1^H *J*_IS_ coupling active during the two spin-echo (*τ*–π–*τ*) durations, *τ*_1_ and *τ*_2_:

1 (NH)  sin(2π*J*_IS_*τ*_2_)sin(2π*J*_IS_*τ*_1_)

*i.e.*, this predicts maximum transfer, for sin(π/2), *i.e.*, *τ* = 1/(4*J*_IS_), *i.e.*, 2.7 ms, for a one-bond ^15^N–^1^H scalar coupling (~90 Hz) for fast MAS alone. When the proton magnetization is along the transverse plane, for example as *Î*_*y*_*Ŝ*_*z*_ during *τ*_2_, the ^1^H–^1^H dipolar couplings shorten the coherence lifetime compared to when the ^1^H magnetization is longitudinal, as during *τ*_1_.^39^ As expanded upon below, the different influence of the interactions is evident in the optimum length of the *τ*_1_ and *τ*_2_ periods: the spectrum in Fig. 1b was recorded with *τ*_2_ (1.0 ms) shorter than *τ*_1_ (2.1 ms); as discussed further below, note that ^1^H homonuclear decoupling scales the *J* coupling.^79–81^

Analogously to the case of ^29^Si–^1^H *J*-couplings in SiH_*n*_ moieties,^82–84^ there is a different dependence on the first spin-echo duration, *τ*_1_, for a NH_3_ moiety:

2 (NH_3_) sin(2π*J*_IS_*τ*_2_)[sin(2π*J*_IS_*τ*_1_) + sin(6π*J*_IS_*τ*_1_)]

As discussed below, a consequence of this is that different signal build-up with respect to *τ*_1_ for a NH and a NH_3_ moiety (and also for a NH_2_ which has a sin(2π*J*_IS_*τ*_2_)sin(4π*J*_IS_*τ*_1_) dependence.

### 3.2 ^1^H PMLG homonuclear decoupling under fast MAS

As noted in the above discussion of Fig. 1a, PMLG ^1^H homonuclear decoupling is employed during the two spin-echo durations of the refocused INEPT pulse sequence element that transfers magnetisation from ^15^N to ^1^H. Lee–Goldburg decoupling^45^ can be considered to be analogous to MAS where the sample is rotated around an axis inclined at the magic angle, *θ*_m_, equal to tan^−1^(√2), to the external magnetic field in that the ratio of the nutation frequency, *ν*_1_, to the resonance offset, Δ*ν*_LG_, is also set equal to tan^−1^(√2). This leads to an effective field, *ν*_eff_LG_, that is given by Pythagoras’ theorem, as:3
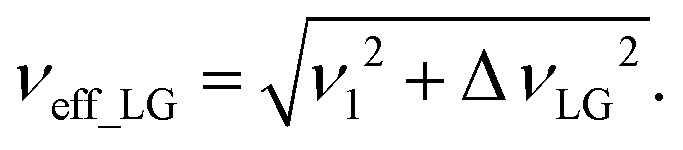
For fixed *ν*_1_, the Lee–Goldburg condition is satisfied as:4
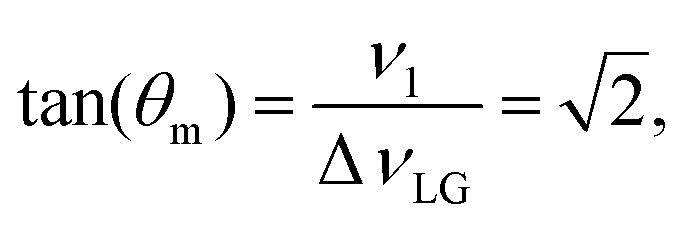
*i.e.*, 
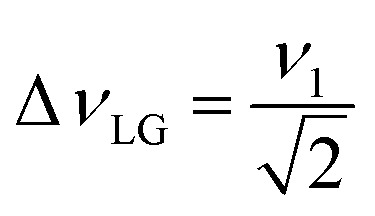
 and 
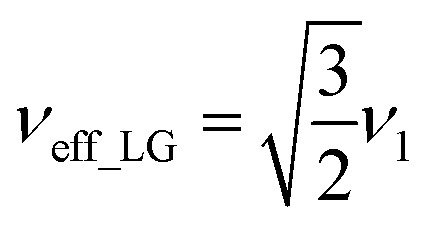
. In the PMLG implementation^49^ of the LG condition, rf irradiation is applied on resonance for a duration, *τ*_LG_, that is the inverse of *ν*_eff_LG_5
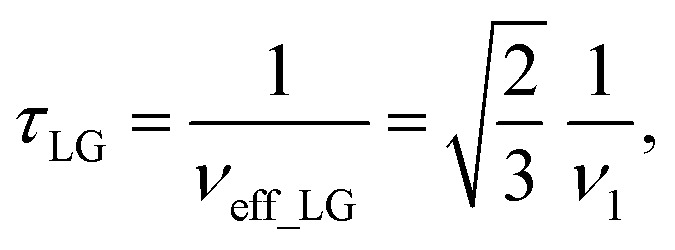
but with an equivalent sweep (in discrete jumps) of the rf phase from 0° to 
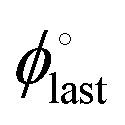
 over the duration, *τ*_LG_, whereby *ϕ*_last_ depends on Δ*ν*_LG_ according to:6
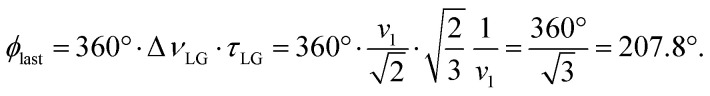
An overall rotation, *ξ*_LG_, of 360° around the effective field is achieved:

7 *ξ*_LG_ = 360°·*ν*_eff_LG_·*τ*_LG_ = 360°.

In the experimental implementation of PMLG under MAS, the duration over which the phase is swept (as discrete steps) from 0° to the ideal *ϕ*_last_ value of 207.8°, *τ*_LG_expt_, can vary from the ideal value, *τ*_LG_. In this way, the equivalent resonance offset, Δ*ν*_expt_, changes from the ideal value, Δ*ν*_LG_, to satisfy: 
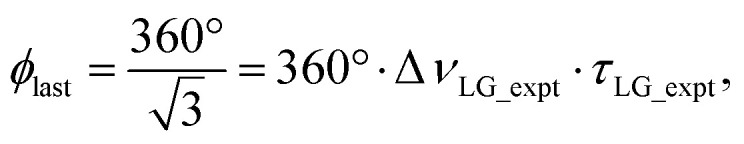
 so that 
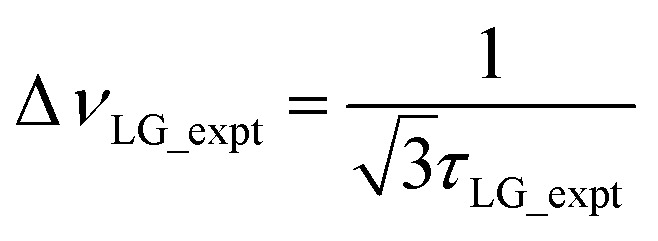
.

Nishiyama *et al.*^57^ have shown that this deviation from the ideal condition can be expressed in terms of how the angle, *θ*, deviates from the magic angle, *θ*_m_:8
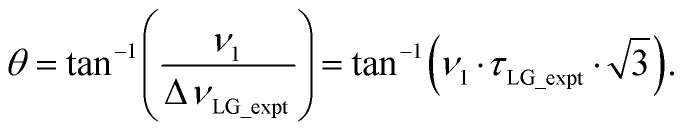
The actual effective field, *ν*_eff_LG_expt_, that is calculated by Pythagoras’ theorem as √(*ν*_1_^2^ + Δ*ν*_LG_expt_^2^) is not equal to 1/*τ*_LG_expt_ and also deviates from the ideal value, *ν*_eff_LG_. As a consequence, the overall rotation about the actual effective field, *ξ*_LG_expt_, also deviates from *ξ*_LG_ = 360° according to:9
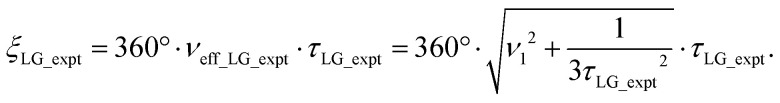
Note that Nishiyama *et al.* refer to this rotation angle as *Ψ*, but this symbol is used in this paper to denote the ratio of the rotor period to the cycle time (see later discussion), according to Leskes *et al.*^65^

**Fig. 2:**
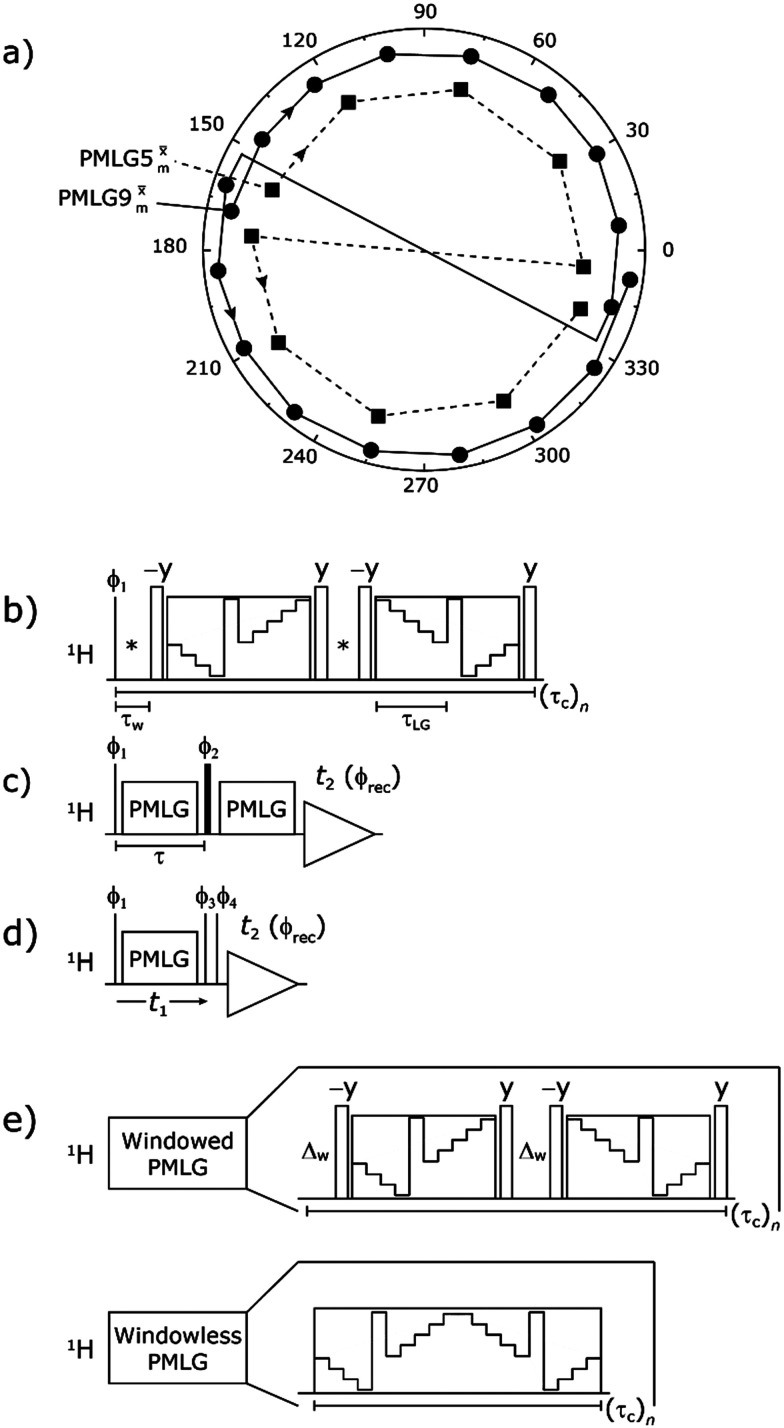
(a) Representation of the phase rotation for 
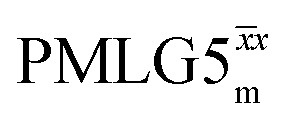
 (dashed line, squares) and 
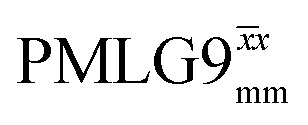
 (solid line, circles). The phase increments are calculated according to *ϕ*_last_ = 207.8° (see eqn (6)), divided by the number of steps. The starting point for both is −*x*. Pulse sequence for (b) a ^1^H 1D CRAMPS experiment with supercycled 
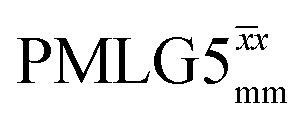
, where the asterisk represents an acquisition window, *τ*_w_, (c) a ^1^H spin-echo and (d) a 2D ^1^H–^1^H correlation experiment. Thin lines and filled rectangles represent 90° and 180° pulses, respectively, while open rectangles denote tilt pulses. In (c) and (d), the block named PMLG can accommodate either (a and e) windowed, where *τ*_w_ is an equivalent period of free evolution, or a windowless sequence, whereby there is continuous rf irradiation during 
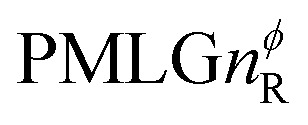
 blocks, *i.e.*, there are no tilt pulses and *τ*_w_ = 0. The following phase cycle is applied for (b) 1D CRAMPS: *ϕ*_1_ = {*x*, −*x*, −*x*, *x*}, *ϕ*_PMLG_ = {*x*, −*x*, −*x*, *x*} and acquisition *ϕ*_rec_ = {*x*, −*x*, −*x*, *x*}; (c) ^1^H spin-echo: *ϕ*_1_ = {*x*, −*x*}, *ϕ*_2_ = {y*2, x*2}, *ϕ*_PMLG_ = {*x*, −*x*} and acquisition *ϕ*_rec_ = {*x*, −*x*, −*x*, *x*}; (d) ^1^H–^1^H homonuclear correlation*: ϕ*_1_ = {*x*, −*x*}, *ϕ*_3_ = {−x*2, x*2}, *ϕ*_4_ = {x*4, y*4}, *ϕ*_PMLG_ = {*x*, −*x*} and acquisition *ϕ*_rec_ = {*x*, −*x*, −*x*, *x*, *y*, −*y*, −*y*, *y*}.

Following the notation of Leskes *et al.*^85^ a PMLG block is specified as 
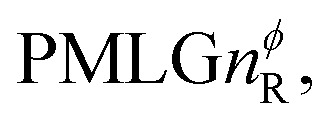
 where: first, *n* is the number of finite pulses for each LG cycle, with *n* equal to 5 or 9 investigated here; second, R is the sense of the initial rotation for the phase steps, *m* for clockwise and *p* for counter-clockwise; and third, the initial phase, *ϕ*, is usually *x* or −*x* (denoted *x̄*). As stated above (see eqn (7)) and as shown in Fig. 2a and b, *τ*_LG_expt_ is the time to sweep the phase over *n* discrete steps, *i.e.*, as *n* finite pulses, from 0° to 207.8°. A single PMLG block, 
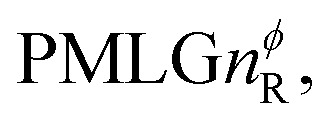
 is of duration 2*τ*_LG_ with a 180° jump after *n* finite pulses in the first *τ*_LG_ followed by *n* finite pulses in the second *τ*_LG_, whereby the phase steps are in the opposite direction. This corresponds to changing the sign of the equivalent resonance offset, as in the frequency-switched (FS) LG experiment, where rf irradiation is alternated between +Δ*ν*_LG_ and −Δ*ν*_LG_.^46,86,87^ As further shown by Leskes *et al.*^85^ supercycling can be achieved as 
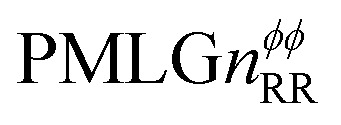
. Specifically, in this work, we use the 
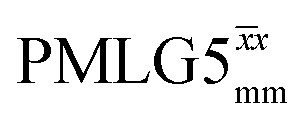
 and 
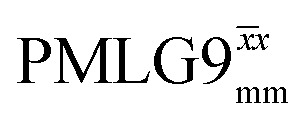
 implementations.

In the windowed implementation of PMLG^88^ acquisition windows of duration *τ*_W_ are placed between the 
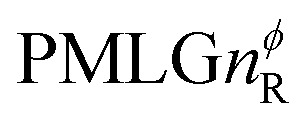
 blocks (see Fig. 2b and e). In addition, tilt pulses of duration *τ*_tilt_ can be used.^53,54,89–91^ The cycle time for a complete 
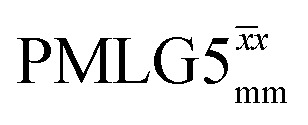
 or 
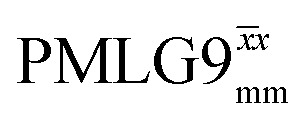
 supercycle, *τ*_c_, is:

10 *τ*_c_ = 2*τ*_w_ + 4*τ*_LG_expt_ + 4*τ*_tilt_.

### 3.3 Optimisation of CH_2_ and NH_3_ signal intensity in a 1D CRAMPS experiment of ^15^N-glycine

**Fig. 3:**
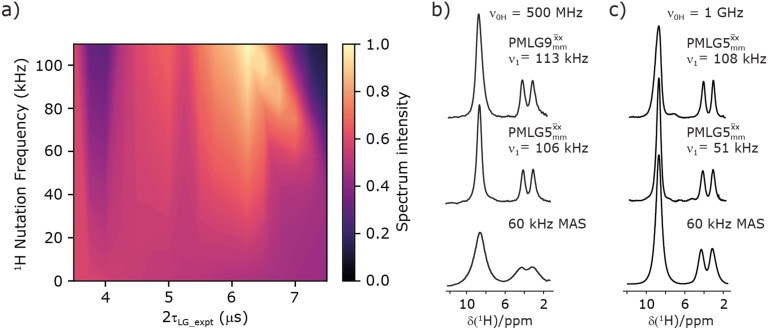
^1^H MAS (*ν*_r_ = 60 kHz) NMR of ^15^N-labelled glycine. (a) 
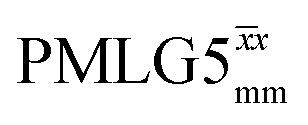
 1D CRAMPS (see Fig. 2b, *τ*_tilt_ = 0.54 μs, *Ω* = −0.6 kHz) two-variable optimization (*ν*_0_ = 500 MHz) of both *τ*_LG_expt_ (in steps of 0.25 μs) and the ^1^H nutation frequency, *ν*_1_ (0–110 kHz) for the NH_3_^+^ peak intensity. (b) Comparison between ^1^H (*ν*_0_ = 500 MHz) 1D CRAMPS MAS NMR spectra acquired with windowed 
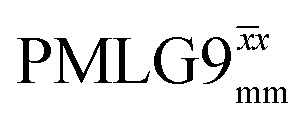
 (*ν*_1_ = 113 kHz, *τ*_LG_expt_ = 2.92 μs, *τ*_tilt_ = 0.82 μs, *Ω* = −0.6 kHz), windowed 
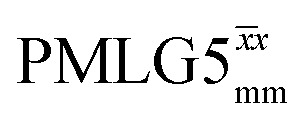
 (*ν*_1_ = 106 kHz, *τ*_LG_expt_ = 3.1 μs, *τ*_tilt_ = 0.54 μs, *Ω* = −0.6 kHz), and a one-pulse MAS-alone experiment. (c) Comparison between ^1^H (*ν*_0_ = 1 GHz) 1D CRAMPS MAS NMR spectra acquired with windowed 
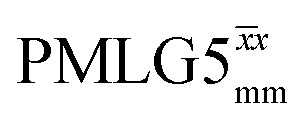
 (*ν*_1_ = 108 kHz, *τ*_LG_expt_ = 3.10 μs, *τ*_tilt_ = 0.18 μs, *Ω* = −7.0 kHz), windowed 
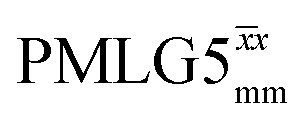
 (*ν*_1_ = 52 kHz, *τ*_LG_expt_ = 3.63 μs, *τ*_tilt_ = 0.70 μs, *Ω* = −8.6 kHz), and a one-pulse MAS-alone experiment. 8 (a) or 32 (b and c) co-added transients were added for a recycle delay of 3 s. For all experiments, *τ*_w_ = 7.20 μs.

The optimization of the ^1^H nutation frequency and *τ*_LG_expt_ is performed differently for windowless and windowed sequences. In this paper, our focus is on windowed sequences that were optimized with a 1D CRAMPS experiment which gives both the chemical shift scaling factor *λ*_CS_ and the ^1^H linewidths in a few seconds for a particular combination of parameters. Specifically for windowed 
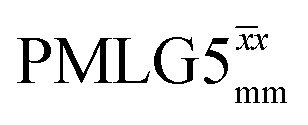
 and 
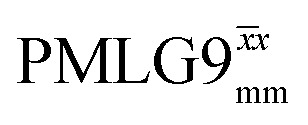
, a two variable optimization was performed over a range of ^1^H nutation frequencies between 0 and 110–120 kHz and *τ*_LG_expt_ between 3.5 and 7.5 μs for ^15^N labelled glycine – see Fig. 3a for 
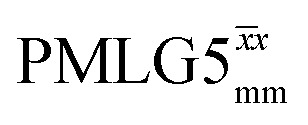
 and Fig. S1 (ESI‡) with slices extracted at different peak intensities, hence with different resolution. (Note that the optimisation of the tilt pulses is discussed in Section S3 of the ESI‡.) For windowless sequences, a coarse optimization was performed, starting from optimised parameters from the 1D CRAMPS experiments, using a ^1^H spin-echo experiment (Fig. 2c) to find good candidate parameters which yield a long ^1^H coherence lifetime. As noted below, the ^1^H–^1^H correlation experiment (Fig. 2d) was used to determine the *λ*_CS_ of the candidate windowless sequences, but can only be used sparingly as the experimental time is relatively long (∼20 minutes for 4 co-added transients and 96 *t*_1_ FIDs for each combination of *τ*_LG_expt_ and *ν*_1_).

Fig. 3a reports on the NH_3_^+ 1^H resonance, noting its relevance in this paper for the ^1^H–^15^N refocused INEPT experiment. Fig. S2 (ESI‡) shows that optimum performance for the NH_3_^+ 1^H resonance (Fig. S2b, ESI‡) is closely matched by that for the CH_2_^1^H resonances (Fig. S2a, ESI‡). 1D CRAMPS ^1^H NMR spectra of ^15^N-glycine for our best implementations of supercycled windowed 
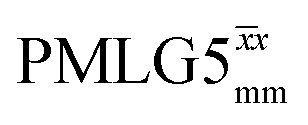
 and 
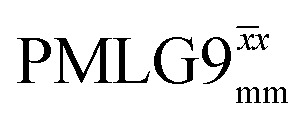
 at *ν*_0_ = 500 MHz are shown in Fig. 3b, where enhanced resolution compared to MAS alone is evident. Moreover, both 
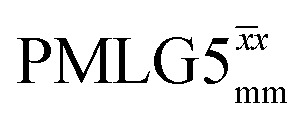
 and 
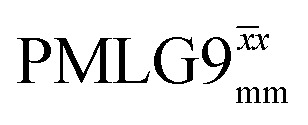
 implemented at *ν*_0_ = 500 MHz (Fig. 3b) show better resolution than 60 kHz MAS alone at *ν*_0_ = 1 GHz (Fig. 3c). At *ν*_0_ = 1 GHz, optimised 1D CRAMPS ^1^H NMR spectra of ^15^N-glycine for windowed 
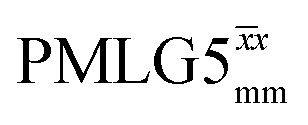
 at a ^1^H nutation frequency of 108 and 51 kHz are presented in Fig. 3c that show enhanced resolution compared to MAS alone. Note that the latter case corresponds to the nutation frequency being less than the MAS frequency.

Table 1 compares the experimentally optimised *τ*_LG_expt_ values to the ideal *τ*_LG_ values: at *ν*_0_ = 500 MHz, the experimental values are less than half the ideal values, *i.e.*, *τ*_LG_expt_ = 3.10 μs and 2.92 μs compared to 7.70 μs and 7.23 μs, respectively. As Table 1 further shows, with the corresponding changes in Δ*ν*_LG_expt_ and *ν*_eff_expt_, the angle *θ* is 29.7°. While a very high nutation frequency of over 200 kHz has been used in the first experimental implementations of PMLG at 65 kHz MAS frequency^59,65^ resulting in a *θ* value of 61° for the spectrum presented by Leskes *et al.*,^59^ a similar value (of 31.2°) far from the magic angle has been reported by Nishiyama *et al.* for the implementation of windowed 
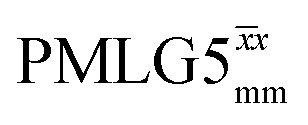
 at an MAS frequency of 80 kHz and a ^1^H nutation frequency of 125 kHz.^57^ Moreover, the actual rotation, *ξ*_LG_expt_, reported by Nishiyama *et al.* of 243° is similar to that of 239° for our implementation of both windowed 
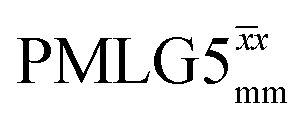
 and 
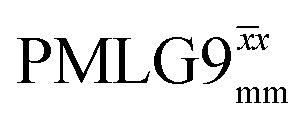
 at a MAS frequency of 60 kHz (see Table 1). Table 1 also lists the implementations of 
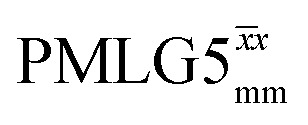
 by Leskes *et al.* at 10 kHz MAS^85^ and Mao & Pruski at 12.5, 19.5, 25.0 and 41.7 kHz MAS:^92^ the angle *θ* is seen to vary between 45° and 64°. It is observed that an angle *θ* below and above the magic angle corresponds to an actual rotation, *ξ*_LG_expt_, less than and more than the ideal 360°, respectively. For the good decoupling performance observed at *ν*_0_ = 1 GHz with windowed 
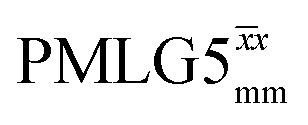
 for a ^1^H nutation frequency of only 51 kHz (see Fig. 3c), the angle *θ* is only 17.6°.

**Table 1:** Implementation of 
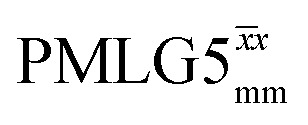
 and 
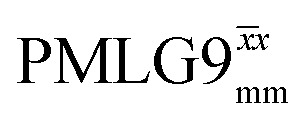
^1^H homonuclear decoupling: variation from the ideal Lee–Goldburg condition for this work and previous publications

Decoupling	*ν*_r_ (kHz)	*ν*_1_ (kHz)	*τ*_LG_ (μs)	*τ*_LG_expt_ (μs)	*θ*_m_ (deg)	*θ* (deg)	Δ*ν*_LG_ (kHz)	Δ*ν*_LG_expt_ (kHz)	*ν*_eff_LG_ (kHz)	*ν*_eff_LG_expt_ (kHz)	*ξ*_LG_ (deg)	*ξ*_LG_expt_ (deg)
Windowed 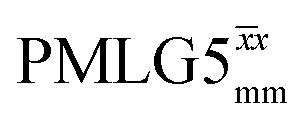 a (500 MHz)	60.0	106	7.70	3.10	54.7	29.7	75.0	186.2	129.8	214.3	360.0	239.2
Windowless 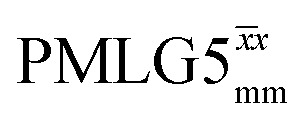 b (500 MHz)	60.0	106	7.70	3.10		29.7	75.0	186.2	129.8	214.3		239.2
Windowed 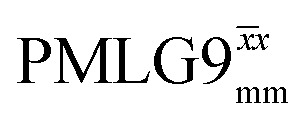 a (500 MHz)	60.0	113	7.23	2.92		29.7	79.9	197.7	138.4	227.7		239.4
Windowless 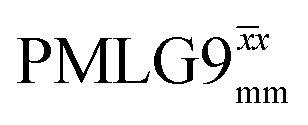 b (500 MHz)	60.0	113	7.23	2.92		29.7	79.9	197.7	138.4	227.7		239.4
Windowed 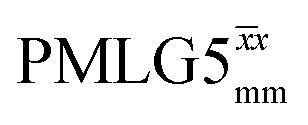 c (1 GHz, *ν*_1_ = 108 kHz)	60.0	108	7.56	3.10		30.1	76.4	186.2	132.3	215.3		240.3
Windowed 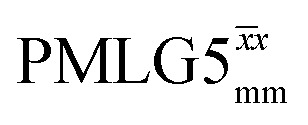 c (1 GHz, *ν*_1_ = 51 kHz)	60.0	51	16.01	3.63		17.6	36.1	159.3	62.4	167.2		218.2

Literature parameters												
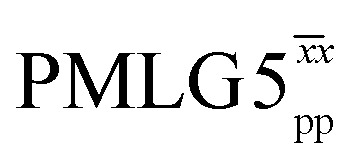 d	80.0	125	6.53	2.80	54.7	31.2	88.4	206.2	153.1	241.1	360.0	243.1
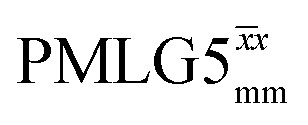 e	65.0	216	3.78	4.80		60.9	152.7	120.3	264.5	247.2		427.2
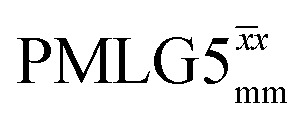 f	41.7	155	5.27	3.75		45.2	109.6	154.0	189.8	218.5		294.9
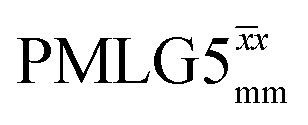 f	41.7	155	5.27	7.75		64.3	109.6	74.5	189.8	172.0		479.8
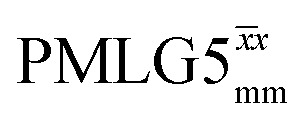 f	12.5	78	10.47	12.50		59.4	55.2	46.2	95.5	90.6		407.9
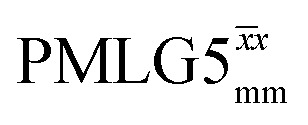 f	19.5	126	6.48	8.00		60.2	89.1	72.2	154.3	145.2		418.2
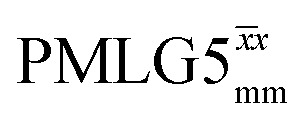 f	25.0	162	5.04	6.25		60.3	114.6	92.4	198.4	186.5		419.6
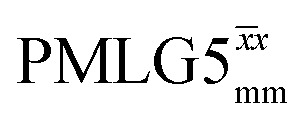 g	10.0	95	8.59	7.25		50.0	67.2	79.6	116.4	124.0		323.5
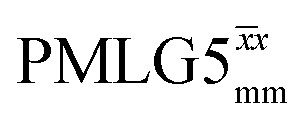 h	65.0	250	3.27	5.00		65.2	176.8	115.5	306.2	275.4		495.7

a Parameters from this work for Fig. 3b and Table 3.

b Parameters from this work for Fig. S3 (ESI).

c Parameters from this work for Fig. 3c and Table 3.

d Values extracted from Nishiyama *et al.* Fig. 2 and 3.^57^

e Values extracted from Leskes *et al.* Table 1.^59^

f Values extracted from Mao and Pruski,^92^ Fig. 3 and 2.

g Values extracted from Leskes *et al.* Fig. 2.^85^

h Simulated values extracted from Leskes *et al.* Fig. 2.^65^

Table 2 states the *τ*_c_ values, as calculated from *τ*_LG_expt_, *τ*_w_ and *τ*_tilt_ using eqn (10), for the implementations of 
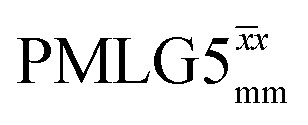
 and 
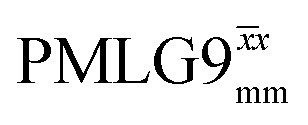
 in this work, as well as that reported in the literature. An important parameter for predicting decoupling performance is the ratio, *Ψ*, of the MAS rotor period, *τ*_r_, to the decoupling cycle time, *τ*_c_, and *vice versa*, the ratio of the corresponding frequency, *ν*_c_ = 1/*τ*_c_, to the MAS frequency, *ν*_r_:^65^11
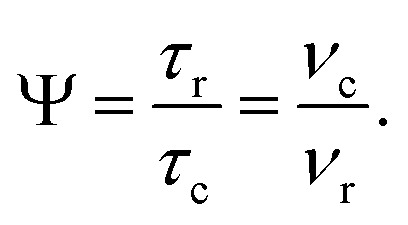
For low to moderate MAS frequencies, small integer values of *Ψ* are to be avoided since these values correspond to recoupling rather than decoupling conditions.^53,91,93–95^ For fast MAS (of at least 40 kHz), there are more values of *Ψ* that need to be avoided.^62,65,92^ Specifically, by employing bimodal Floquet theory, Leskes *et al.* have identified values of *n* and *k* that result in deteriorated decoupling due to zero-order and first-order recoupling conditions, according to:

12 *nν*_r_ + *kν*_r_ = 0,

where *n* takes values 1, 2, 3, 4 while −15 ≤ *k* ≤ −1.^65^ While there is a dense set of degeneracies for values of *Ψ* below 1.50, there are windows of good decoupling performance that can be found. The *Ψ* value of both the windowless sequences, 
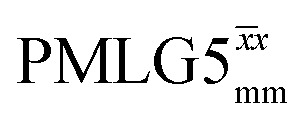
 (*Ψ* = 1.34) and 
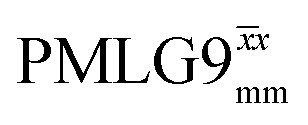
 (*Ψ* = 1.43), are in line with the value of 1.40–1.60 reported by Mao *et al.* (in Tables 1 and 2 of their paper) for spectra acquired among a range of different spinning frequencies (12.5 kHz to 41.7 kHz) and ^1^H nutation frequencies (78–162 kHz).^92^ For windowed sequences, the *Ψ* value is usually lower. For the 1D CRAMPS spectra presented in Fig. 3b, Table 2 shows that *Ψ* equals 0.58 and 0.57 for windowed 
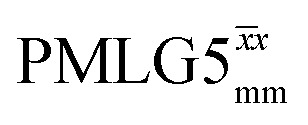
 and windowed 
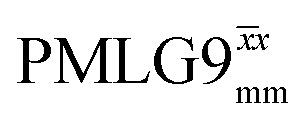
, respectively, at *ν*_0_ = 500 MHz, and 0.61 and 0.53 at *ν*_0_ = 1 GHz for a ^1^H nutation frequency of 108 and 51 kHz, respectively. These *Ψ* values are similar to the values of 0.60 and 0.63 for the experimental implementation of windowed 
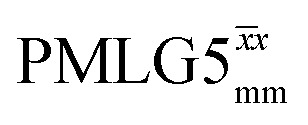
 by Nishiyama *et al.* at an MAS frequency of 80 kHz and a ^1^H nutation frequency of 125 kHz^57^ and by Leskes *et al.* at an MAS frequency of 65 kHz and a ^1^H nutation frequency of 216 kHz,^59^ respectively.

**Table 2:** Implementation of 
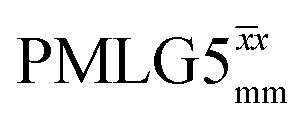
 and 
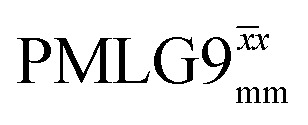
^1^H homonuclear decoupling: scaling factors and comparison of rotor period to cycle time for this work and previous publications

	*τ*_LG_expt_ (μs)	*τ*_w_ (μs)	*τ*_tilt_ (μs)	*τ*_c_ (μs)	*τ*_r_ (μs)	*Ψ* k	*λ*_CS_calc_	*λ*_CS_expt_
Windowed 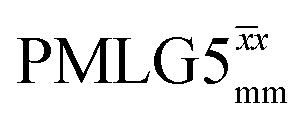 a (500 MHz)	3.10	7.20	0.54	28.96	16.67	0.58	0.76^j^	0.82
Windowless 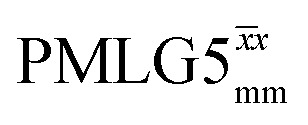 b (500 MHz)	3.10	—	—	12.40	16.67	1.34	0.76^i^	0.66
Windowed 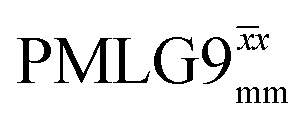 a (500 MHz)	2.92	7.20	0.82	29.36	16.67	0.57	0.77^j^	0.76
Windowless 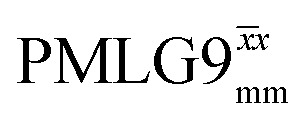 b (500 MHz)	2.92	—	—	11.68	16.67	1.43	0.78^i^	0.60
Windowed 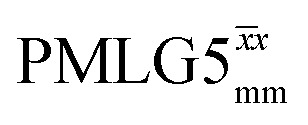 c (1 GHz, 108 kHz)	3.10	7.20	0.18	27.52	16.67	0.61	0.74^j^	0.82
Windowed 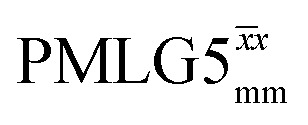 c (1 GHz, 51 kHz)	3.63	7.20	0.70	31.70	16.67	0.53	0.90^j^	0.92

Literature parameters								
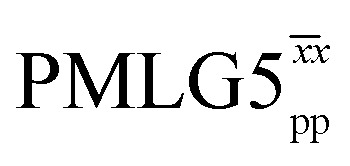 d	2.80	4.84	—	20.88	12.50	0.60	0.86^i^	0.82
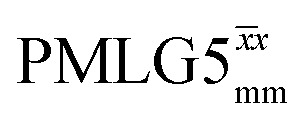 e	4.80	2.70	—	24.60	15.38	0.63	0.40^i^	0.48
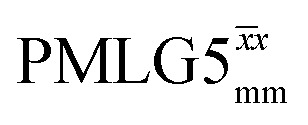 f	3.75	—	—	15.00	24.00	1.60	0.50^i^	0.36
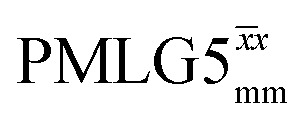 f	7.75	—	—	31.00	24.00	0.77	0.19^i^	0.21
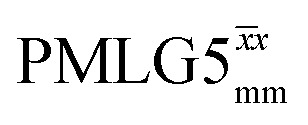 f	12.50	—	—	50.00	80.00	1.60	0.26^i^	—
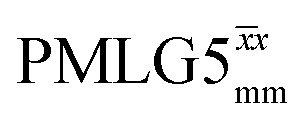 f	8.00	—	—	32.00	51.20	1.60	0.25^i^	—
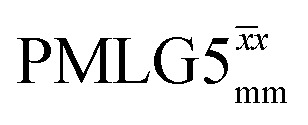 f	6.25	—	—	25.00	40.00	1.60	0.25^i^	—
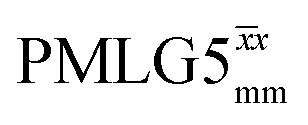 g	7.25	4.35	—	37.70	100.00	2.65	0.55^i^	0.47
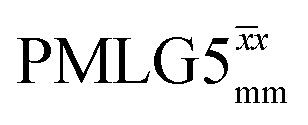 h	5.00	—	—	20.00	15.38	0.77	0.18^i^	—

a Parameters from this work for Fig. 3b and Table 3.

b Parameters from this work for Fig. S5 (ESI).

c Parameters from this work for Fig. 3c and Table 3.

d Values extracted from Nishiyama *et al.* Fig. 2 and 3.^57^

e Values extracted from Leskes *et al.* Table 1.^59^

f Values extracted from Mao and Pruski,^92^ Fig. 3 and 2.

g Values extracted from Leskes *et al.* Fig. 2.^85^

h Simulated values extracted from Leskes *et al.* Fig. 2.^65^

i *λ*_CS_ is calculated with eqn (15) as stated in this paper, following from Nishiyama *et al.*^57^

j *λ*_CS_ is calculated with eqn (16) as stated in this paper, following from Nishiyama *et al.*^57^

k *Ψ* is calculated with eqn (12), following from Leskes *et al.*^65^

### 3.4 Windowed and windowless PMLG ^1^H decoupling, ^1^H spin-echo dephasing and scaling factors

It is well established that the application of rf ^1^H homonuclear decoupling leads to a chemical shift scaling: for a static sample, the chemical shift scaling factor, *λ*_CS_, for perfect decoupling cannot exceed cos^−1^(*θ*_m_) = 1/√3 = 0.577.^64,95,96^ The 1D ^1^H CRAMPS spectra presented in Fig. 3b and c have chemical shift axes that have been corrected for this scaling, *i.e.*, a scaling is applied so as to ensure that the chemical shift separation between the NH_3_^+^ peak and the lower ppm CH_2_ peak corresponds to the MAS-only ^1^H chemical shifts, *i.e.*, 8.4–3.0 = 5.4 ppm. The full width at half maximum, (FWHM), of the three ^1^H resonances before and after scaling for the spectra presented in Fig. 3b and c are presented in Table 3. Table 3 also states that *λ*_CS_ equals 0.82 and 0.76 for windowed 
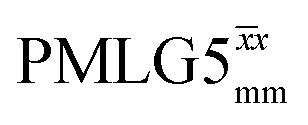
 and windowed 
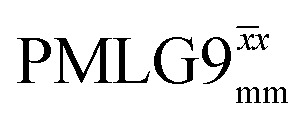
, respectively, at *ν*_0_ = 500 MHz, and 0.82 and 0.92 at *ν*_0_ = 1 GHz for a ^1^H nutation frequency of 108 and 51 kHz, respectively. Table 3 also reports, as a measure of decoupling efficiency, *K*, given by13
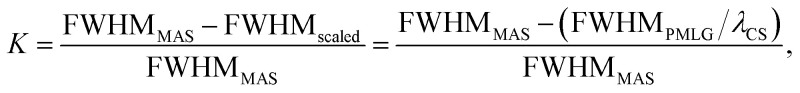
where a *K* closer to 1 corresponds to better decoupling performance. FWHM_MAS_ is obtained under MAS alone, FWHM_PMLG_ is the linewidth recorded using PMLG, and FWHM after scaling, FWHM_scaled_, is equal to FWHM_PMLG_/*λ*_CS_. High scaling factors that are significantly above 0.577, like those stated in Table 3, have been reported for 60 kHz MAS by Salager *et al.* for an experimental optimisation protocol based on a quality factor considering the intensity of the two most intense resonances, CH_3_ and NH_3_, in β-AspAla as well as their peak separation in Hz.^58^ Specifically, *λ*_CS_ equals 0.73 and 0.84 for the eDUMBO-PLUS-1 and eDUMBO-PLUS-large sequences, respectively, for 60 kHz MAS and a ^1^H nutation frequency of 170 kHz, with optimum resolution observed for eDUMBO-PLUS-1. Salager *et al.* have further presented a scaling factor theorem for homonuclear decoupling, derived for a static system of homonuclear *I* = 1/2 spins coupled by a dipolar interaction that are subject to cyclic rf irradiation:14
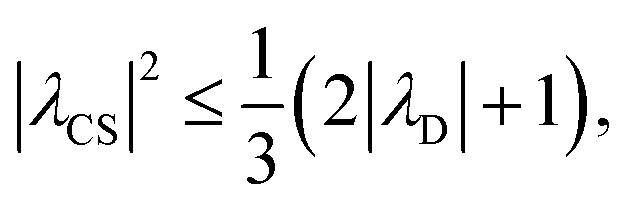
where *λ*_D_ is the dipolar scaling factor, *i.e.*, zero corresponds to perfect decoupling, showing that *λ*_CS_ cannot exceed 1/√3, when *λ*_D_ = 0.^64^

**Table 3:** Analysis of windowed 
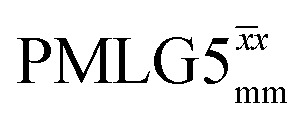
 and 
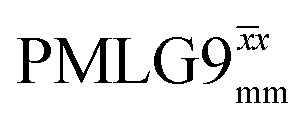
^1^H homonuclear decoupling efficiency for ^1^H (*ν*_0_ = 500 MHz and 1 GHz) CRAMPS NMR at *ν*_r_ = 60 kHz of ^15^N-glycine^a^

	δ (ppm)	FWHM_MAS_ (Hz)	FWHM_MAS_ (ppm)	FWHM_PMLG_ (Hz)	FWHM_PMLG_ (ppm)	FWHM_scaled_ (Hz)	FWHM_scaled_ (ppm)	Scaling factor, *λ*_CS_	*K* b	FWHM_PMLG_ (Hz)	FWHM_PMLG_ (ppm)	FWHM_scaled_ (Hz)	FWHM_scaled_ (ppm)	Scaling factor, *λ*_CS_	*K* b
*ν*_0_ = 500 MHz				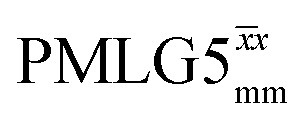 (*ν*_1_ = 106 kHz)						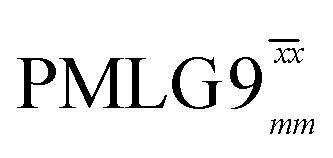 (*ν*_1_ = 113 kHz)					
NH_3_^+^	8.4	664	1.33	230	0.46	280	0.56	0.82	0.58	273	0.55	359	0.72	0.76	0.46
CH_2_	4.2	800^c^	1.60	217	0.43	264	0.53		0.67	213	0.43	280	0.56		0.65
CH_2_	3.0	800^c^	1.60	224	0.45	273	0.55		0.66	232	0.46	305	0.61		0.62

*ν*_0_ = 1 GHz				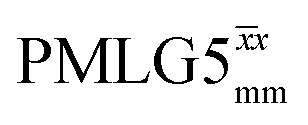 (*ν*_1_ = 108 kHz)						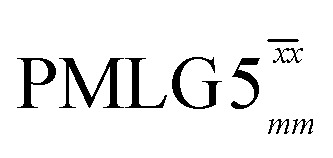 (*ν*_1_ = 51 kHz)					
NH_3_^+^	8.4	700	0.70	583	0.58	711	0.71	0.82	−0.02	475	0.48	516	0.52	0.92	0.26
CH_2_	4.2	740	0.74	346	0.35	422	0.42		0.43	448	0.45	487	0.49		0.34
CH_2_	3.0	740	0.74	311	0.31	379	0.38		0.49	440	0.44	478	0.48		0.35

a See spectra in Fig. 3b (*ν*_0_ = 500 MHz) and Fig. 3c (*ν*_0_ = 1 GHz), for the pulse sequence in Fig. 2b and experimental parameters in Table 2.

b Calculated with eqn (13).

c FWHM extracted from the indirect dimension of a 2D ^1^H–^1^H correlation experiment with MAS alone, see Fig. S4 in the ESI.

For 
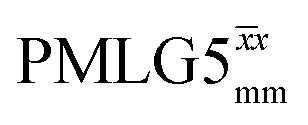
, Nishiyama *et al.* report a *λ*_CS_ of 0.82 at 80 kHz MAS and a ^1^H nutation frequency of 125 kHz. Nishiyama *et al.* further state equations for calculating *λ*_CS_ for 
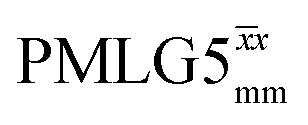
 decoupling without and with tilt pulses:15
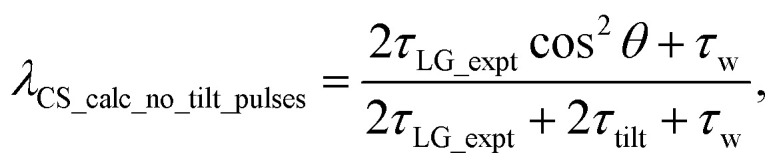
16
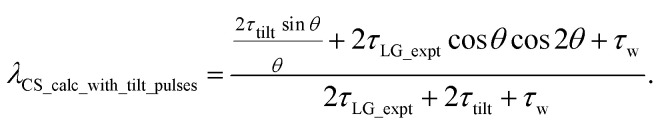
These calculated *λ*_CS_ values are presented in Table 2 for the experimental implementations of 
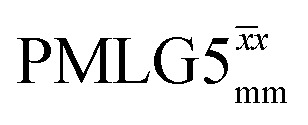
 in the literature, as well as 
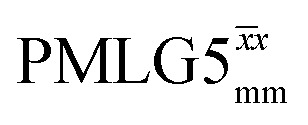
 and 
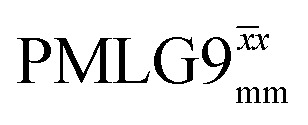
 in this work. Deviation of the experimental scaling factor compared to theoretical behaviour can arise from phase transients that cause phase propagation delays.^91,97^

As well as scaling the chemical shifts, ^1^H homonuclear decoupling also scales evolution under a heteronuclear *J* coupling by the same factor.^37,57,79^ For magnetisation transfer from ^15^N to ^1^H during the spin echoes of the refocused INEPT pulse sequence element, the efficiency depends upon this scaling of the ^15^N–^1^H *J* couplings, but also the spin-echo dephasing time, 
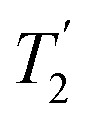
.^92,98,99^

**Fig. 4:**
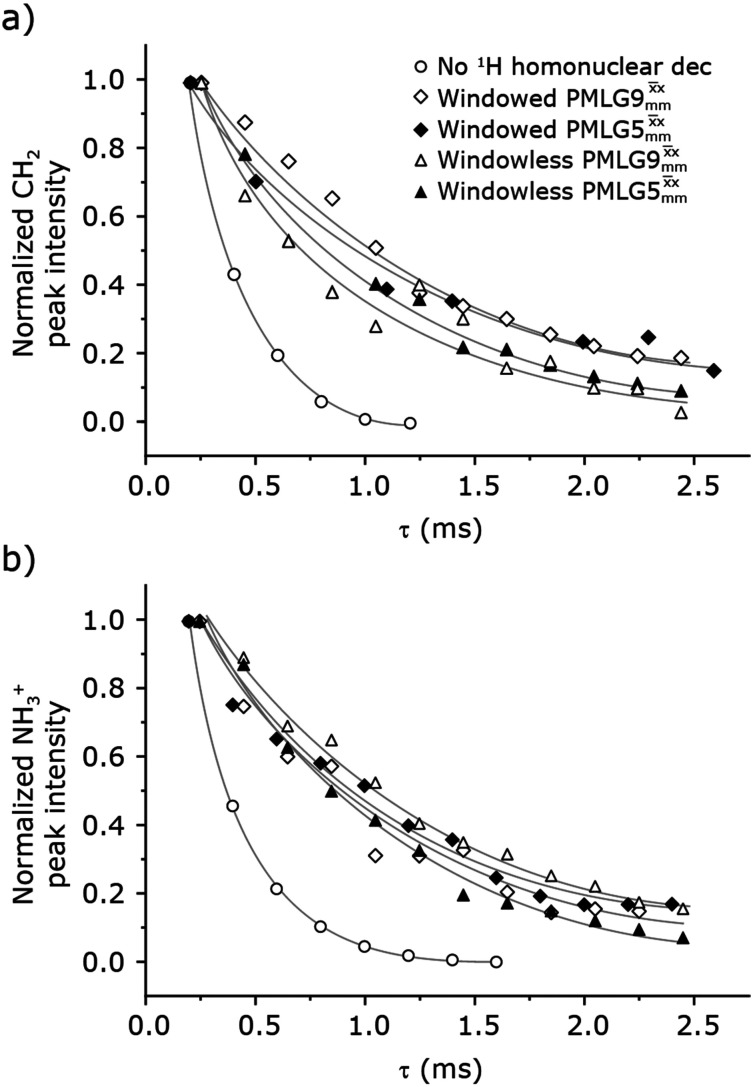
Dephasing of the ^15^N-glycine (a) CH_2_ (the higher ppm ^1^H resonance is considered) and (b) NH_3_^+^ proton resonances as a function of the spin-echo (see Fig. 2c) duration, *τ*, with no ^1^H homonuclear decoupling (empty circles), windowed 
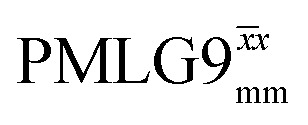
(empty diamonds), windowed 
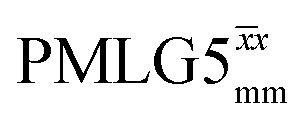
 (full diamonds), windowless 
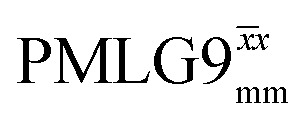
 (empty triangles), and windowless 
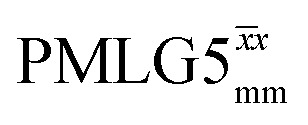
 (full triangles) for nutation frequencies and resonance offsets as stated in Table 4. Fits to an exponential decay function are shown, with the spin-echo dephasing times, 
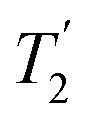
, as listed in Table 4. 16 transients were co-added for a recycle delay of 3 s. For all experiments with windowed ^1^H homonuclear decoupling, *τ*_w_ = 7.20 μs.

Fig. 4 compares spin-echo dephasing curves (see pulse sequence in Fig. 2c) for MAS alone to those for windowed and windowless 
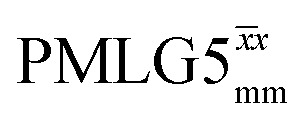
 and 
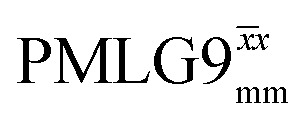
, with the values for experimental parameters and extracted 
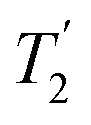
 presented in Table 4. (Note that 
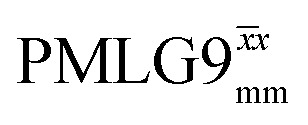
 homonuclear decoupling was implemented with a slightly changed nutation frequency of *ν*_1_ = 109 kHz, as compared to *ν*_1_ = 113 kHz for the 1D CRAMPS spectrum in Fig. 3b). In windowless PMLG decoupling, there is continuous rf irradiation, *i.e.*, there are no tilt pulses and *τ*_w_ = 0, while, in the windowed version, *τ*_w_ is replaced by a delay (Fig. 2e.) Note that the first implementation of PMLG was in the indirect dimension of a two-dimensional ^1^H–^1^H experiment where there is evolution under MAS alone in the direct dimension.^49^ Such a 2D experiment (see Fig. 2d) is used to measure *λ*_CS_ for our implementation of windowless 
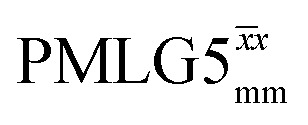
 and 
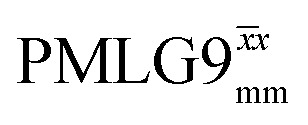
, as reported in Tables 2 and 4 (spectra are presented in Fig. S4, ESI‡).

Considering Fig. 4 and Table 4, the ^1^H dephasing times, 
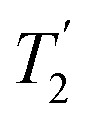
, for the CH_2_ (the higher ppm resonance is considered) and NH_3_^+^ peaks are 0.22 ms and 0.25 ms for 60 kHz MAS alone. With ^1^H homonuclear decoupling the ^1^H dephasing time for both groups increases. The longest CH_2_ dephasing time is observed for windowed 
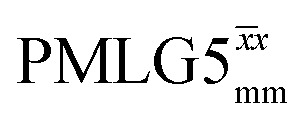
, 
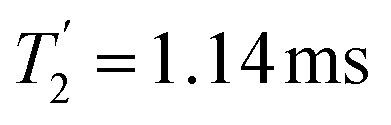
, slightly longer than for windowed 
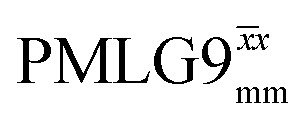
, where 
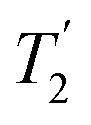
 is equal to 1.10 ms. However, the scaling by *λ*_CS_ needs to be considered and Table 4 reports the product of *λ*_CS_ and 
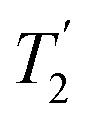
 in each case. After this scaling (Table 4), windowed 
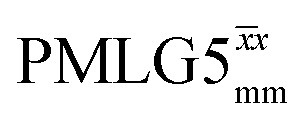
 achieves an over 4 fold improvement with respect of MAS alone, compared to the slightly under 4 fold improvement of windowed 
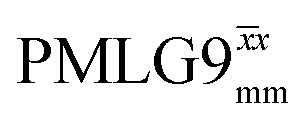
. A similar comparison can be made for the NH_3_^+^ peak, where windowless 
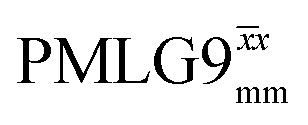
 shows the longest 
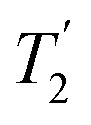
 equal to 1.15 ms and the longest value of the product, *λ*_CS_
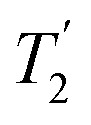
 of 0.69 ms, thanks again to the large *λ*_CS_; this corresponds to a just under 3 fold improvement with respect to MAS alone.

### 3.5 Optimisation of the ^15^N-glycine NH_3_^+^ signal intensity in a 1D-filtered CP-refocused INEPT NMR spectrum for PMLG ^1^H decoupling at 60 kHz MAS

**Fig. 5:**
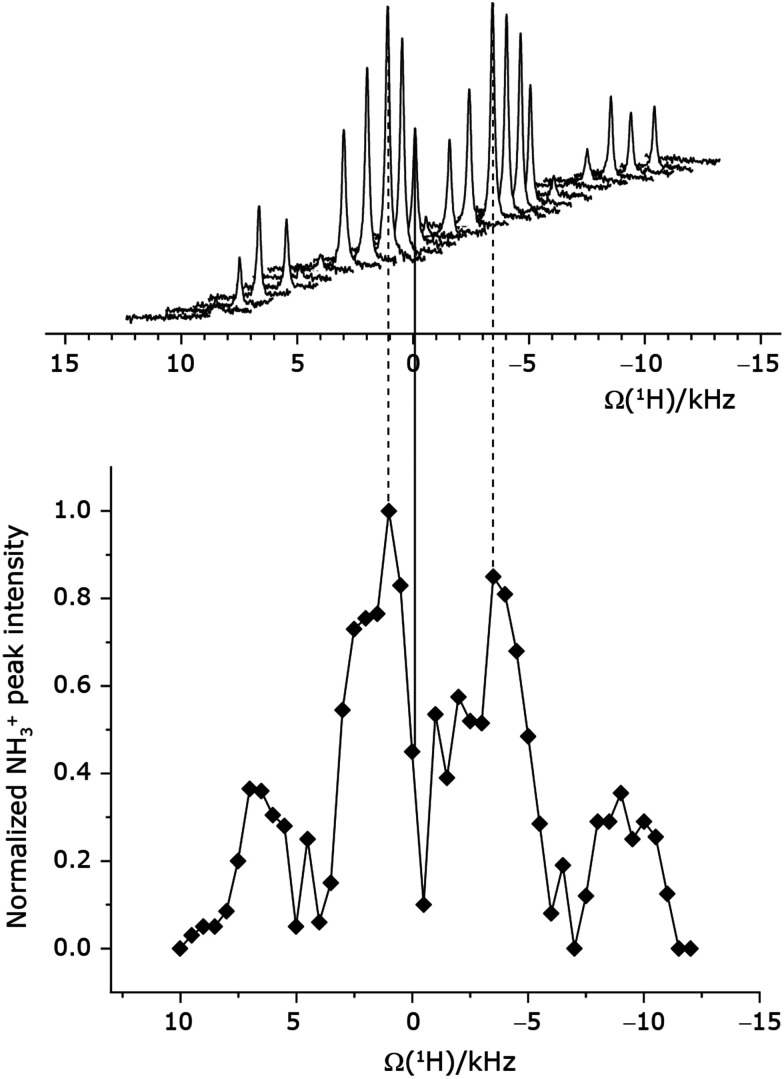
^1^H RF carrier optimization for a 1D-filtered (*t*_1_ = 0) ^15^N–^1^H (*ν*_0_ = 500 MHz) CP (contact time = 2 ms)-refocused INEPT MAS (*ν*_r_ = 60 kHz) NMR experiment for ^15^N-labelled glycine, whereby windowed 
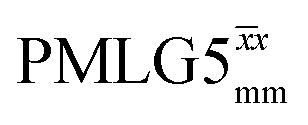
^1^H homonuclear decoupling was applied with *τ*_LG_expt_ = 3.1 μs, *τ*_tilt_ = 0.54 μs and a ^1^H nutation frequency, *ν*_1_, of 106 kHz during *τ*_1_ (1.999 ms, 69*τ*_c_) and 104 kHz during *τ*_2_ (1.391 ms, 48*τ*_c_). 16 transients were coadded. For all experiments with windowed ^1^H homonuclear decoupling, *τ*_w_ = 7.20 μs. The zero-offset is set with the carrier being on resonance with the NH_3_^+^ peak, corresponding to the solid vertical line. Dashed vertical lines indicate the two highest signal intensities at +1 kHz and −3.5 kHz.

Under a ^1^H homonuclear decoupling sequence such as PMLG, the proton offset frequency influences the performance;^53,54^ this is linked to the overall *z*-rotation that the spins need under decoupling to avoid artifacts and RF imperfections.^85^ As shown by Leskes *et al.*,^89^ the non-supercycled m-block is particularly beneficial in narrowing lines of strong coupled spins, as for the CH_2_ groups of ^15^N-glycine, close to the on-resonance position. With the implementation of supercycled PMLG schemes,^90^ the sign of the offset is no longer a determining factor as the supercycle brings the effective rotation of the spins closer to the *z*-axis.^100^ However, the choice of the optimum offset still plays a significant role for achieving good decoupling performance, therefore it is necessary to investigate both positive and negative offsets. Here the optimization was performed directly on the ^15^N–^1^H CP-refocused INEPT experiment, where windowed 
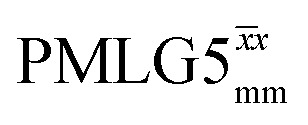
 was applied over a wide range of offset values from ∼+10 kHz to −12 kHz, whereby on-resonance corresponds to the NH_3_^+^ peak. Fig. 5 shows that the best offsets in term of sensitivity are at +1 kHz and −3.5 kHz, highlighted by dashed vertical lines. Between the two best performing offsets, the sensitivity experiences a fluctuation (Fig. 5) corresponding to the on-resonance position (solid line), dropping to zero for a small negative offset of −0.5 kHz. It is then important to optimize the offset avoiding the on-resonance position. The need for a fine optimization of this parameter is emphasized by the considerable change in sensitivity that is observed for a small variation of the offset.^53,54,95^ For example, the relative sensitivity of the NH_3_^+^ peak falls from over 0.8 to 0.5 when switching the offset from ∼−3.5 to −2.5 kHz. In general, in Fig. 5 the offsets close to the on-resonance position yield better sensitivity symmetrically in a range between ±4 kHz, in agreement with the rotation improvement brought by the supercycled ^1^H homonuclear decoupling.^89^

The same offset optimization was carried out on the different PMLG-block types, and similar trends were shown with a better sensitivity in the proximity of the on-resonance position. As stated in Table 4, the offsets which gave the maximum sensitivity were 0.75 kHz for windowed 
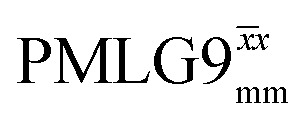
, −0.25 kHz for 
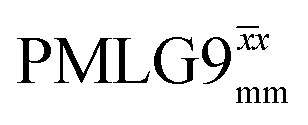
 and +1 kHz for 
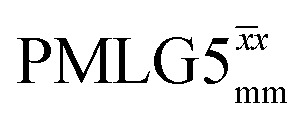
 (the same as windowed 
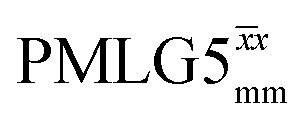
) (see Fig. S5, ESI‡).

**Table 4:** ^1^H dephasing time, 
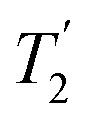
, and 
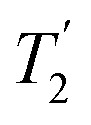
 scaled by the experimental *λ*_CS_, *λ*_CS_
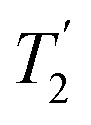
, as determined by a ^1^H spin-echo MAS NMR experiment^a^ for ^15^N-glycine with optimised rf carrier offset and *ν*_1_

	Offset (kHz)	*ν*_1_ (kHz)	*λ*_CS_	NH_3_^+^ 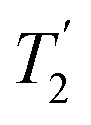 (ms)	NH_3_^+^*λ*_CS_ 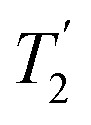 (ms)	CH_2_ 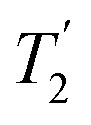 b (ms)	CH_2_*λ*_CS_ 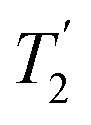 (ms)
No decoupling	2	—	1	0.25	0.25	0.22	0.22
Windowed 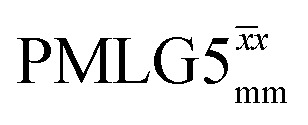	1	106	0.82	1.04	0.85	1.14	0.93
Windowed 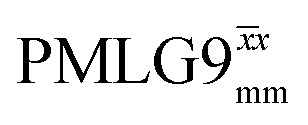	0.75	109	0.76	0.91	0.69	1.10	0.84
Windowless 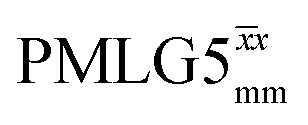	1	106	0.66	0.86	0.57	0.80	0.53
Windowless 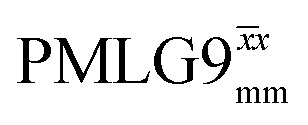	−0.25	109	0.60	1.15	0.69	0.78	0.47

a aAs implemented at *ν*_0_ = 500 MHz and *ν*_r_ = 60 kHz, see Fig. 4a for the CH_2_ resonance and Fig. 4b for the NH_3_^+^ peak. *τ*_tilt_ is equal to 0.54 μs for windowed 
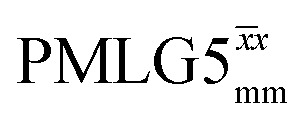
 and 0.82 μs for windowed 
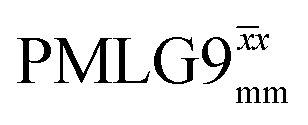
.

b For the CH_2_ group, the 
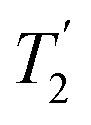
 of the higher-ppm ^1^H resonance is stated.

**Fig. 6:**
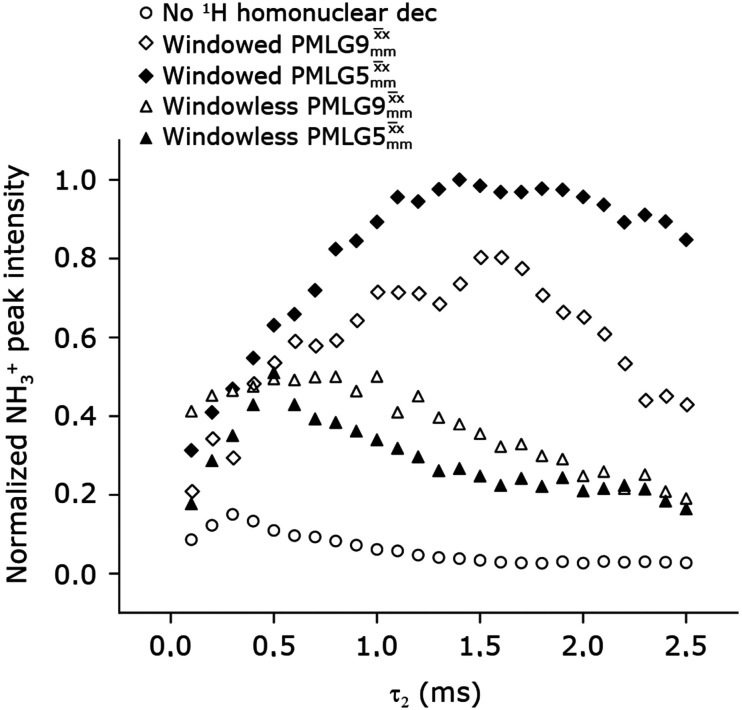
Dependence upon the second spin-echo duration, *τ*_2_, for ^15^N-labelled glycine of the NH_3_^+^ peak in a 1D-filtered (*t*_1_ = 0) ^15^N–^1^H (*ν*_0_ = 500 MHz) CP (contact time = 2 ms)-refocused INEPT MAS (*ν*_r_ = 60 kHz) NMR spectrum for: windowed 
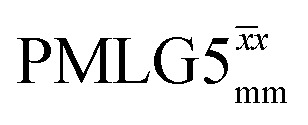
 (*τ*_LG*_*expt_ = 3.1 μs, *τ*_tilt_ = 0.54 μs, *ν*_1_ = 106 kHz for *τ*_1_ and 106 kHz for *τ*_2_ full diamonds), windowless 
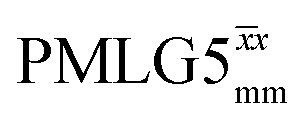
 (same conditions but with no tilt pulses, full triangles, with *ν*_1_ = 102 kHz for *τ*_1_ and 102 kHz for *τ*_2_), windowed 
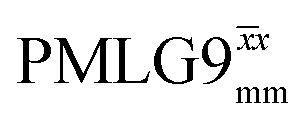
 (*τ*_LG_expt_ = 2.92 μs, *τ*_tilt_ = 0.82 μs, *ν*_1_ = 104 kHz for *τ*_1_ and 106 kHz for *τ*_2_ empty diamonds), windowless 
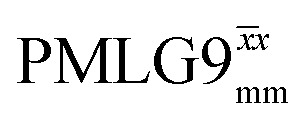
 (same conditions but with no tilt pulses, empty triangles, with *ν*_1_ = 104 kHz for *τ*_1_ and 102 kHz for *τ*_2_), MAS alone (empty circles). 8 transients were coadded. For all experiments with windowed PMLG, *τ*_w_ = 7.20 μs.

The implementation of the ^1^H decoupling scheme into the heteronuclear correlation experiment required the further optimisation of the spin-echo durations during the refocused INEPT transfer. This was carried out separately for *τ*_1_ and *τ*_2_ (see pulse sequence in Fig. 1a) because, as stated in Section 3.1, for the two spin echoes, different spins are along the transverse plane, ^15^N for the first and ^1^H for the second spin echo. To ensure the best conditions, a double-optimisation of ^1^H homonuclear decoupling nutation frequency *vs. τ*_1_ and *τ*_2_ was carried out. Specifically, the two-variable optimisation was performed for ^15^N-labelled glycine for windowed or windowless 
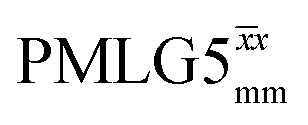
 and 
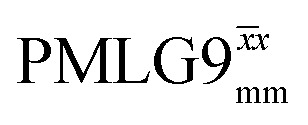
 for the best offset (see Table 5) and the results are reported in Table 5. The dependence with respect to the second spin-echo duration, *τ*_2_, is presented in Fig. 6. Note from eqn (2), a sine dependence is expected from which the scaled *J* coupling could be extracted.

**Table 5:** Optimised rf carrier offset, spin-echo duration and nutation frequencies for four implementations of PMLG ^1^H homonuclear decoupling and MAS-alone for a ^15^N–^1^H CP-refocused INEPT MAS NMR experiment for ^15^N-glycine^a^

^1^H homonuclear decoupling	Offset^b^ (kHz)	*λ*_CS_	*τ*_1_ c (ms)	*λ*_CS_ *τ*_1_ (ms)	*ν*_1_ (kHz) for *τ*_1_	*τ*_2_ c (ms)	*λ*_CS_ *τ*_2_ (ms)	*ν*_1_ (kHz) for *τ*_2_	Relative intensity^d^
No decoupling	2.00	1.00	1.600	1.600	—	0.300	0.300	—	0.08
Windowed 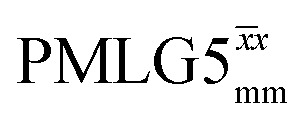	1.00	0.82	1.999 (69*τ*_c_)	1.639	106	1.391 (48*τ*_c_)	1.140	106	1.00
Windowed 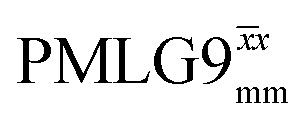	0.75	0.76	2.085 (71*τ*_c_)	1.585	104	1.498 (51*τ*_c_)	1.138	106	0.80
Windowless 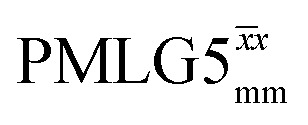	1.00	0.66	2.096 (169*τ*_c_)	1.383	102	0.496 (40*τ*_c_)	0.327	102	0.52
Windowless 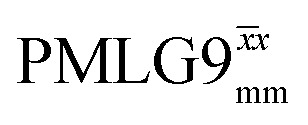	−0.25	0.60	2.091 (179*τ*_c_)	1.254	104	1.192 (102*τ*_c_)	0.715	102	0.48

a As implemented at *ν*_0_ = 500 MHz and *ν*_r_ = 60 kHz. *τ*_tilt_ is equal to 0.54 μs for windowed 
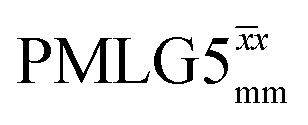
 and 0.82 μs for windowed 
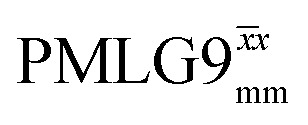
. See Fig. 6.

b Relative to the NH_3_^+ 1^H resonance.

c *τ*_1_ = *nτ*_c_, *τ*_2_ = *mτ*_c_, where *n* and *m* are positive integers.

d See Fig. 7.

Considering Table 5, the ^1^H nutation frequencies are in the range of 102–106 kHz for all the PMLG-block types, with a maximum of 2 kHz difference between that applied in *τ*_1_ and *τ*_2_ for the same PMLG block. For *τ*_1_, the optimum values for PMLG decoupling are 2.0 or 2.1 ms, as compared to 1.6 ms from MAS alone. However, as discussed in Section 3.4, it is the product *λ*_CS_·*τ*, that needs to be considered, in which case similar values are obtained as compared to MAS alone. By comparison, a clear difference is observed for *τ*_2_, where the evolution of ^1^H coherence is markedly affected by the ^1^H–^1^H dipolar couplings. Indeed, the coherence transfer increases from 0.3 ms for MAS alone to 1.5 ms for windowed 
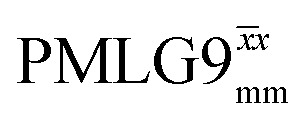
 and 1.4 ms for windowed 
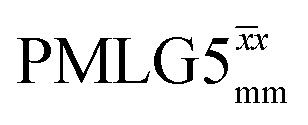
. After scaling, the product *λ*_CS_*τ*_2_, 1.14 ms for both windowed 
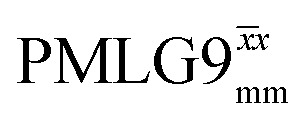
 and 
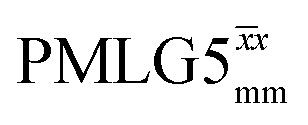
, are still ∼4 times longer than the optimum *τ*_2_ for MAS alone. We note a discrepancy for *τ*_2_ under windowless 
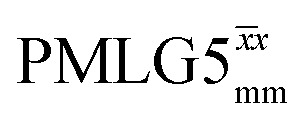
, which is considerably shorter (0.3 ms after scaling) with respect to the other ^1^H homonuclear implementations.

**Fig. 7:**
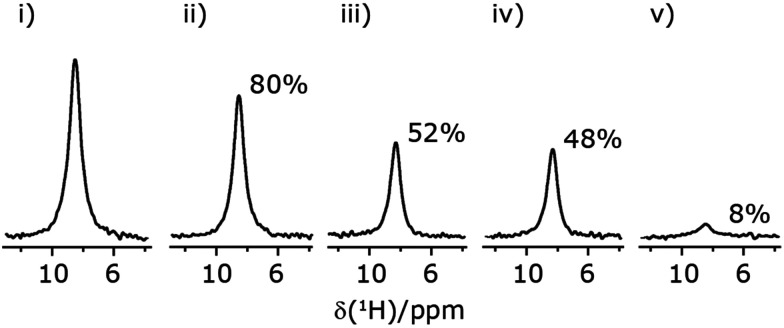
Comparison of the sensitivity of 1D-filtered (*t*_1_ = 0) ^15^N–^1^H (*ν*_0_ = 500 MHz) CP (contact time = 2 ms)-refocused INEPT MAS (*ν*_r_ = 60 kHz) NMR spectra of ^15^N-glycine recorded with the application of different optimised PMLG ^1^H decoupling conditions, (i)–(iv) compared to MAS alone, (v): (i) windowed 
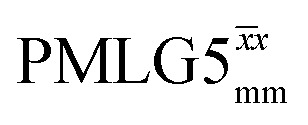
 (*τ*_LG*_*expt_ = 3.1 μs, *τ*_tilt_ = 0.54 μs, *τ*_1_ = 1.999 ms (69*τ*_c_) with *ν*_1_ = 106 kHz; *τ*_2_ = 1.391 ms (48*τ*_c_) with *ν*_1_ = 106 kHz), (ii) windowed 
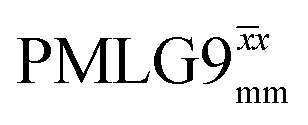
 (*τ*_LG_expt_ = 2.92 μs, *τ*_tilt_ = 0.82 μs, *τ*_1_ = 2.085 ms (71*τ*_c_) with *ν*_1_ = 104 kHz; *τ*_2_ = 1.498 ms (51*τ*_c_) with *ν*_1_ = 106 kHz), (iii) windowless 
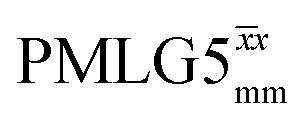
 (*τ*_LG_expt_ = 3.1 μs, *τ*_1_ = 2.096 ms (169*τ*_c_) with *ν*_1_ = 102 kHz; *τ*_2_ = 0.496 ms (40*τ*_c_) with *ν*_1_ = 102 kHz), (iv) windowless 
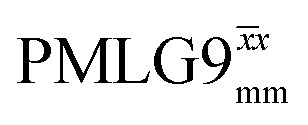
 (*τ*_LG_expt_ = 2.92 μs, *τ*_1_ = 2.090 ms (179*τ*_c_) with *ν*_1_ = 104 kHz*; τ*_2_ = 1.192 ms (102*τ*_c_) with *ν*_1_ = 102 kHz), (v) no decoupling *τ*_1_ = 1.6 ms (96*τ*_r_) and *τ*_2_ = 0.3 ms (18*τ*_r_). For all experiments with windowed ^1^H homonuclear decoupling, *τ*_w_ = 7.20 μs. All the spectra were acquired with 16 coadded transients and the corresponding ^1^H transmitter offset reported in Table 5.

In Fig. 7, we compare the different peak intensities for the NH_3_^+^ peak of ^15^N-labelled glycine for the windowless and windowed implementation of 
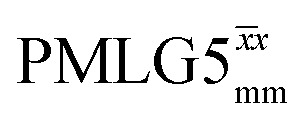
 and 
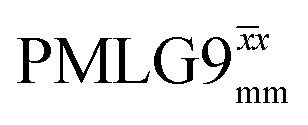
 in a ^15^N–^1^H CP-refocused INEPT 1D filtered (*t*_1_ = 0) spectrum. The best performance is for our optimum implementation of windowed 
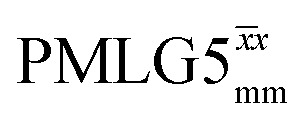
 with a 12.5 times better relative sensitivity compared to MAS alone.

**Fig. 8:**
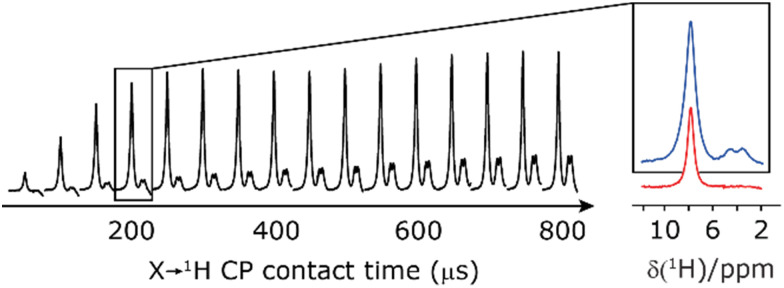
Comparison of the sensitivity of 1D-filtered (*t*_1_ = 0) ^15^N–^1^H (*ν*_0_ = 600 MHz) MAS (*ν*_r_ = 60 kHz) NMR spectra of ^15^N-glycine recorded with a double CP experiment (blue) or a CP-refocused INEPT experiment (red). The build-up for the double CP experiment as a function of the ^15^N to ^1^H CP contact time is also shown. In both cases, the ^1^H to ^15^N CP contact time is 3.7 ms. For refocused INEPT ^15^N to ^1^H transfer, windowed 
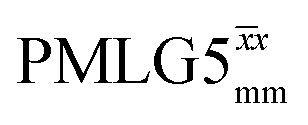
 (*τ*_LG_expt_ = 3.19 μs, *τ*_tilt_ = 0.5 μs and *τ*_w_ = 7.20 μs) is applied at a nutation frequency of 106 kHz for *τ*_1_ = 2.334 ms (140*τ*_r_) and *τ*_2_ = 1.401 ms (84*τ*_r_). All the spectra were acquired with 16 co-added transients and a ^1^H transmitter offset of −4 kHz.

Finally, in this section, we compare the sensitivity and selectivity of the CP refocused INEPT experiment to that of a hNH double CP experiment. Specifically, the right-hand side of Fig. 8 compares 1D-filtered MAS NMR spectra of ^15^N-glycine recorded using the CP refocused INEPT experiment (red) or a hNH double CP experiment with a back (^15^N to ^1^H) CP contact time of 200 μs (blue). In both cases, the ^1^H to ^15^N CP contact time is 3.7 ms, *i.e.*, CP is used initially to efficiently generate ^15^N transverse magnetisation. While the sensitivity of the CP refocused INEPT spectrum is half that of the double CP experiment, there is no intensity for the CH_2_^1^H resonances. Fig. 8 also shows, for the double CP experiment, the dependence on the back (^15^N to ^1^H) CP contact time, with a plateau in intensity reached after 200 μs, though note that CH_2_^1^H resonance signal is already evident from 100 μs.

### 3.6 2D ^15^N–^1^H CP-refocused INEPT NMR spectra with PMLG ^1^H decoupling at 60 kHz MAS of a dipeptide and a pharmaceutical at natural abundance

**Fig. 9:**
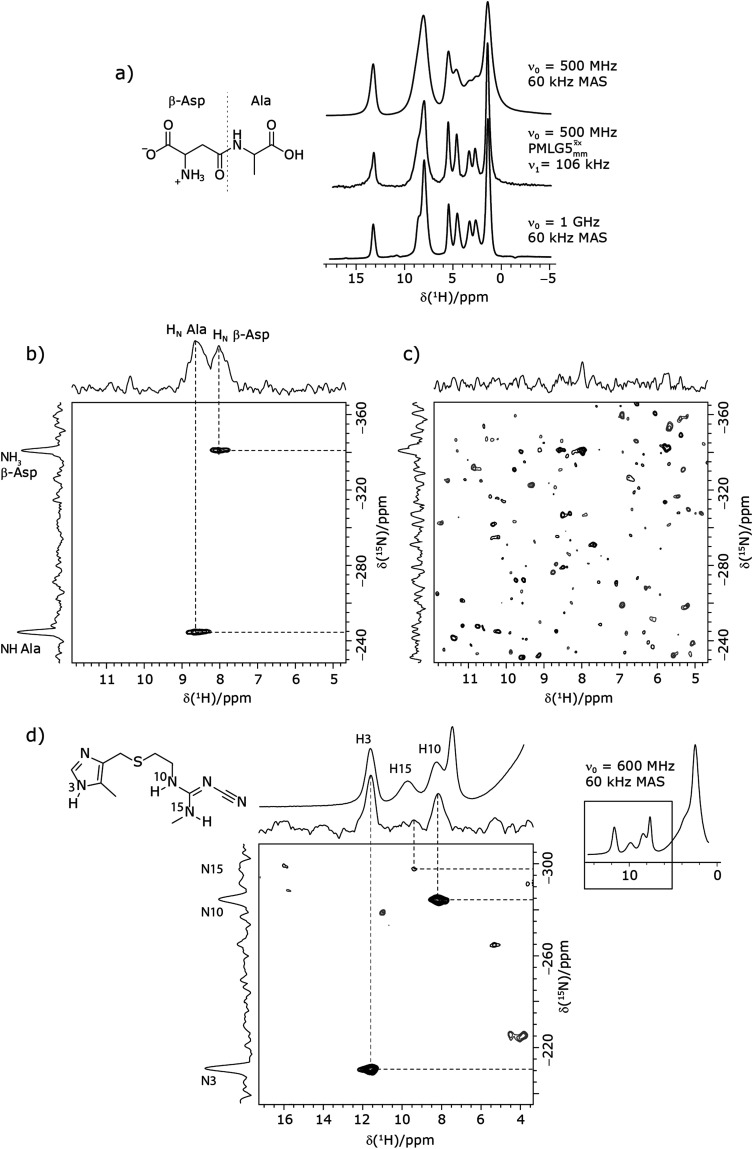
MAS (*ν*_r_ = 60 kHz) NMR spectra of (a–c) the dipeptide β-AspAla and (d) the pharmaceutical cimetidine, in both cases at natural isotopic abundance, employing windowed 
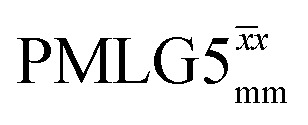
 (*τ*_LG*_*expt_ = 3.1 μs, *τ*_tilt_ = 0.54 μs and *τ*_w_ = 7.20 μs). (a) Comparison of a ^1^H 1D CRAMPS spectrum acquired with windowed 
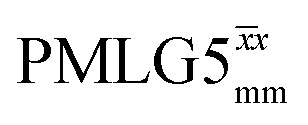
 (at *ν*_0_ = 500 MHz, with ^1^H one-pulse spectra recorded at *ν*_0_ = 500 MHz and 1 GHz. (b and c) 2D ^15^N–^1^H (*ν*_0_ = 500 MHz) CP (contact time = 2 ms)-refocused INEPT MAS NMR spectra with (b) windowed 
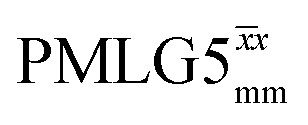
^1^H homonuclear decoupling during the spin-echo durations used for ^15^N–^1^H refocused INEPT coherence transfer or (c) MAS alone. In (b), windowed 
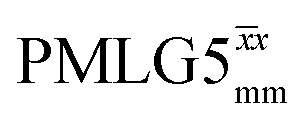
 was implemented with *ν*_1_(^1^H) = 106 kHz during *τ*_1_ (1.999 ms, 69*τ*_c_) and *ν*_1_(^1^H) = 106 kHz during *τ*_2_ (1.391 ms, 48*τ*_c_), with the transmitter frequency centred at 10.3 ppm. For both (b) and (c), 224 transients were co-added for each of 96 *t*_1_ FIDs, corresponding to a total experimental time of 23 h with a recycle delay of 3 s. The base contour is at 50% of the respective maximum intensity in (b) and (c). (d) A 2D ^15^N–^1^H (*ν*_0_ = 600 MHz) CP (contact time = 4 ms)-refocused INEPT MAS NMR spectrum with windowed 
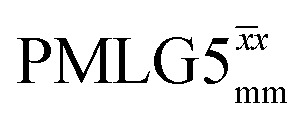
^1^H homonuclear decoupling (*ν*_1_(^1^H) = 106 kHz during *τ*_1_ (2.491 ms, 86*τ*_c_) and *ν*_1_(^1^H) = 106 kHz during *τ*_2_ (1.999 ms, 69*τ*_c_)), with the transmitter frequency centred at 11.0 ppm. 1024 transients were co-added for each of 64*t*_1_ FIDs, corresponding to a total experimental time of 92 h with a recycle of 5 s. The base contour is at 30% of the maximum intensity.

Due to the better sensitivity of windowed 
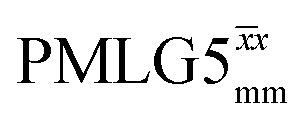
 observed for glycine, it was selected as the ^1^H homonuclear decoupling sequence for a ^15^N–^1^H correlation experiment recorded for the β-AspAla dipeptide at natural isotopic abundance, with the improvement of resolution achieved in the 1D ^1^H CRAMPS compared here with a ^1^H one-pulse recorded at Larmor frequency of 500 MHz and 1 GHz (Fig. 9a). Note that a ^15^N CP MAS spectrum for the β-AspAla dipeptide has been presented in Tatton *et al.*^22^ The ^15^N–^1^H CP-refocused INEPT experiment was implemented with the offset and coherence transfer delays optimised for ^15^N-labelled glycine, as stated in Table 5, *i.e.*, *τ*_LG_expt_ = 3.1 μs, *τ*_tilt_ = 0.54 μs, *τ*_1_ = 2.0 ms with *ν*_1_ = 106 kHz, *v*_2_ = 1.4 ms with *ν*_1_ = 106 kHz, and an offset of +1 kHz. High-performance ^1^H homonuclear decoupling achieved with a finely optimised implementation of windowed 
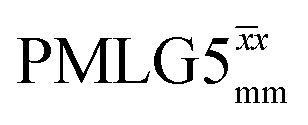
 enables the recording at natural abundance of a 2D ^15^N–^1^H correlation spectrum at 60 kHz MAS with a through-bond back transfer (Fig. 9b). The sensitivity of the windowed 
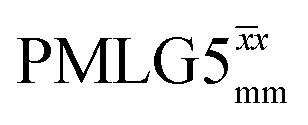
 implementation is compared to a ^15^N–^1^H CP-refocused INEPT spectrum recorded with no decoupling at the optimum *τ*_1_ = 1.6 ms and *τ*_2_ = 0.3 ms values in Table 5 for ^15^N-labelled glycine; only noise is observed in Fig. 9c.

**Fig. 10:**
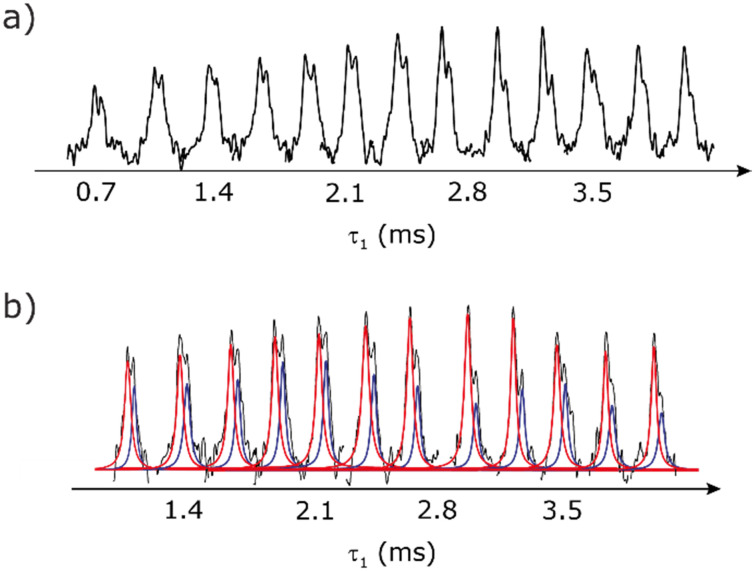
(a) Dependence upon the first spin-echo duration, *τ*_1_, for a 1D-filtered (*t*_1_ = 0) ^15^N–^1^H (*ν*_0_ = 600 MHz) CP (contact time = 3.7 ms)-refocused INEPT MAS (*ν*_r_ = 60 kHz) NMR spectrum for the dipeptide β-AspAla at natural isotopic abundance, recorded using ^1^H homonuclear decoupling (*τ*_LG*_*expt_ = 3.19 μs, *τ*_tilt_ = 0.50 μs, with *ν*_1_ = 106 kHz) for *τ*_2_ = 2.101 ms (126*τ*_r_). All the spectra were acquired with 1024 co-added transients and a ^1^H transmitter offset of −2 kHz. A deconvolution of the NH (red) and NH_3_ (blue) peaks is shown in (b).

As noted in Section 3.1, there is a different dependence on the duration of the first spin echo, *τ*_1_, for a NH and NH_3_^+^ moiety, compare eqn (1) and (2). This is evident from Fig. 10 that shows the build-up of intensity in a 1D-filtered ^15^N–^1^H CP-refocused INEPT spectrum of the dipeptide β-AspAla. Two peaks are resolved for the higher-ppm NH and the lower-ppm NH_3_^+^ resonances (see deconvolution in Fig. 10b), and it is evident maximum intensity is reached at a shorter spin-echo duration for the lower-ppm NH_3_^+^ peak at ∼2.1 ms as compared to ∼3.5 ms for the higher-ppm NH peak. As shown in Fig. S7 of the ESI,‡ this is expected as based from a consideration of eqn (1) and (2). Such an experiment could hence be used to distinguish different NH_*x*_ moieties, as for example has been demonstrated analogously for SiH_*x*_ groups.^82–84^

Furthermore, windowed 
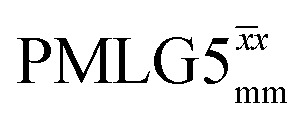
 was employed to record a 2D ^15^N–^1^H CP-refocused INEPT spectrum of the pharmaceutical cimetidine at natural abundance (Fig. 9d), for which ^1^H, ^15^N CPMAS and ^14^N–^1^H spectra have been presented in ref. 101 and 102. (For comparison, note that in ref. 101, Tatton *et al.* use a simple ^15^N–^1^H heteronuclear spin echo with ^1^H homonuclear decoupling to demonstrate spectral editing.) In this case, spin-echo curves were recorded, because, as discussed above, the optimum *τ*_1_ and *τ*_2_ durations in the refocused INEPT pulse sequence element depends both on the *J* coupling between the involved nuclei and the ^1^H dephasing 
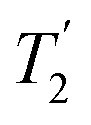
. The ^1^H coherence lifetime (see Fig. S6 and Table S1 (ESI‡) in comparison to Table 4) for two of the protons directly bonded to the nitrogens, N3 and N10, is longer than the NH_3_^+^
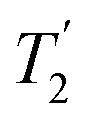
 of ^15^N-glycine acquired with the same windowed 
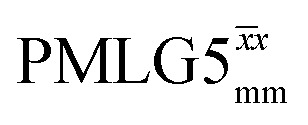
^1^H decoupling. In addition, considering the above discussion of Fig. 10 and eqn (1) and (2), note that for a NH group, a maximum signal is observed at a longer *τ*_1_ as compared to a NH_3_^+^ group. For this reason, *τ*_1_ and *τ*_2_ were increased to 2.5 ms and 2.0 ms, respectively. Note that weaker intensity is observed for the proton directly bonded to N15, where the respective ^1^H 
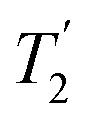
 is ∼0.5 ms after scaling (Table S1, ESI‡). Further investigation is required to understand the shorter 
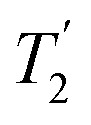
 for this proton and the very weak signal for the N15–H15 cross peak in the 2D CP-refocused INEPT spectrum in Fig. 9d.

## 4. Conclusions and outlook

This paper has identified ^1^H homonuclear decoupling conditions for the 
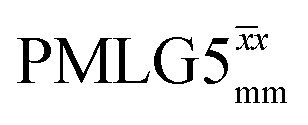
 supercycle at 60 kHz MAS that give enhanced resolution in a 1D NMR spectrum as compared to MAS alone. At 1 GHz, we report what we believe to be the first example of effective homonuclear decoupling achieved by using a rf nutation frequency lower than the MAS frequency. The establishing of 2D ^15^N–^1^H heteronuclear correlation for natural abundance solids using a ^1^H detected CP-*J* coupling based refocused INEPT MAS NMR experiment^26,38,39^ has been demonstrated at 60 kHz MAS. The application of ^1^H homonuclear decoupling, specifically the 
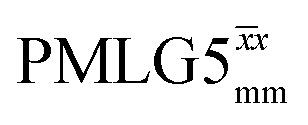
 supercycle^26,39,57,85^ results in a factor of 12.5 sensitivity enhancement as compared to MAS alone. Notably, in our implementation at 500 MHz, a comparatively low ^1^H nutation frequency, for a 1.3 mm rotor, of 100 kHz was used, with this being associated with a high chemical shift scaling factor of 0.82 and a large deviation from the ideal Lee–Goldburg condition. Future work could further probe the suitability and optimisation of such windowed and windowless decoupling sequences for applications involving spin-echo evolution. In addition, nutation-frequency-selective pulses that reduce rf inhomogeneity could also be explored.^103^ The CP-refocused INEPT pulse sequence is complementary to dipolar coupling-based double CP or the use of symmetry-based decoupling to establish ^15^N–^1^H heteronuclear correlation under fast MAS.^26,29,30,104^ Note that the use of symmetry-based recoupling is more prone to *t*_1_ noise.^105–107^ In future work, the extension of our approach to 100+ kHz MAS could be considered, noting an increasing number of applications to pharmaceuticals and other small and moderately sized organic molecules.^9,108–114^

## Conflicts of interest

There are no conflicts to declare.

## Supplementary Material

CP-024-D2CP01041K-s001
