# Correction: Optimisation of ^1^H PMLG homonuclear decoupling at 60 kHz MAS to enable ^15^N–^1^H through-bond heteronuclear correlation solid-state NMR spectroscopy

**DOI:** 10.1039/d2cp90167f

**Published:** 2022-09-13

**Authors:** Jacqueline Tognetti, W. Trent Franks, Józef R. Lewandowski, Steven P. Brown

**Affiliations:** Department of Chemistry, University of Warwick Coventry CV4 7AL UK; Department of Physics, University of Warwick Coventry CV4 7AL UK S.P.Brown@warwick.ac.uk

## Abstract

Correction for ‘Optimisation of ^1^H PMLG homonuclear decoupling at 60 kHz MAS to enable ^15^N–^1^H through-bond heteronuclear correlation solid-state NMR spectroscopy’ by Jacqueline Tognetti *et al.*, *Phys. Chem. Chem. Phys.*, 2022, **24**, 20258–20273, https://doi.org/10.1039/D2CP01041K.

The authors would like to make the following change in the Acknowledgements section of the published article.

‘Data for this study are provided as a supporting data set from the University of Warwick Research Datasets portal at https://wrap.warwick.ac.uk/166703/.’ is amended to ‘Data for this study are provided as a supporting data set from the University of Warwick Research Datasets portal at https://wrap.warwick.ac.uk/167703/’

The Royal Society of Chemistry apologises for these errors and any consequent inconvenience to authors and readers.

## Supplementary Material

