# Supplementary material for: Optimisation of 1H PMLG homonuclear decoupling at 60 kHz MAS to enable 15N–1H through-bond heteronuclear correlation solid-state NMR spectroscopy
Source: Phys Chem Chem Phys. 2022 Jul 26;24(34):20258–73. doi: 10.1039/d2cp01041k (PMC9429863; doi:10.1039/d2cp01041k)
Supplement: CP-024-D2CP01041K-s001 [file CP-024-D2CP01041K-s001.pdf]

Electronic Supplementary Material (ESI) for Physical Chemistry Chemical Physics.
This journal is © the Owner Societies 2022

Electronic Supplementary Information (ESI)

Optimisation of ^{1}H PMLG homonuclear decoupling at 60 kHz MAS to enable ^{15}N-^{1}H through-bond heteronuclear correlation solid-state NMR spectroscopy

Jacqueline Tognetti, W. Trent Franks, Józef R. Lewandowski, Steven P. Brown

S1. Product operator formalism - INEPT

We review here a product operator analysis of the refocused INEPT pulse sequence element. At the beginning of the refocused INEPT element, the in-phase magnetization $\hat{S}_x$ is along the transverse plane for ^{15}N. During the first echo period (τ_1), the in-phase magnetization is converted into anti-phase $\hat{S}_y\hat{I}_z$:

$$\hat{S}_x \xrightarrow{\tau_1-\pi-\tau_1} \cos(2\pi J_{IS}\tau_1)\hat{S}_x + \sin(2\pi J_{IS}\tau_1)\hat{S}_y\hat{I}_z \, , \qquad (1)$$

where $\hat{I}$ represents the ^{1}H spins. The anti-phase coherence is transferred from *S* to *I* with the 90˚ pulses applied on both channels, which separates the two spin-echo evolution periods:

$$\sin(2\pi J_{IS}\tau_1)\hat{S}_y\hat{I}_z \xrightarrow{(\pi/2)\hat{I}_x} \xrightarrow{(\pi/2)\hat{S}_x} \sin(2\pi J_{IS}\tau_1)\hat{S}_z\hat{I}_y \, . \qquad (2)$$

Following τ_1, in the second echo period (τ_2), the antiphase ^{1}H coherence is converted into in-phase $\hat{I}_x$ that is then detected during acquisition (t_2):

$$\sin(2\pi J_{IS}\tau_1)\hat{S}_z\hat{I}_y \xrightarrow{\tau_2-\pi-\tau_2} \sin(2\pi J_{IS}\tau_2)\sin(2\pi J_{IS}\tau_1)\hat{I}_x \, . \qquad (3)$$

S2. Optimisation of PMLG ^{1}H homonuclear decoupling on ^{15}N-glycine

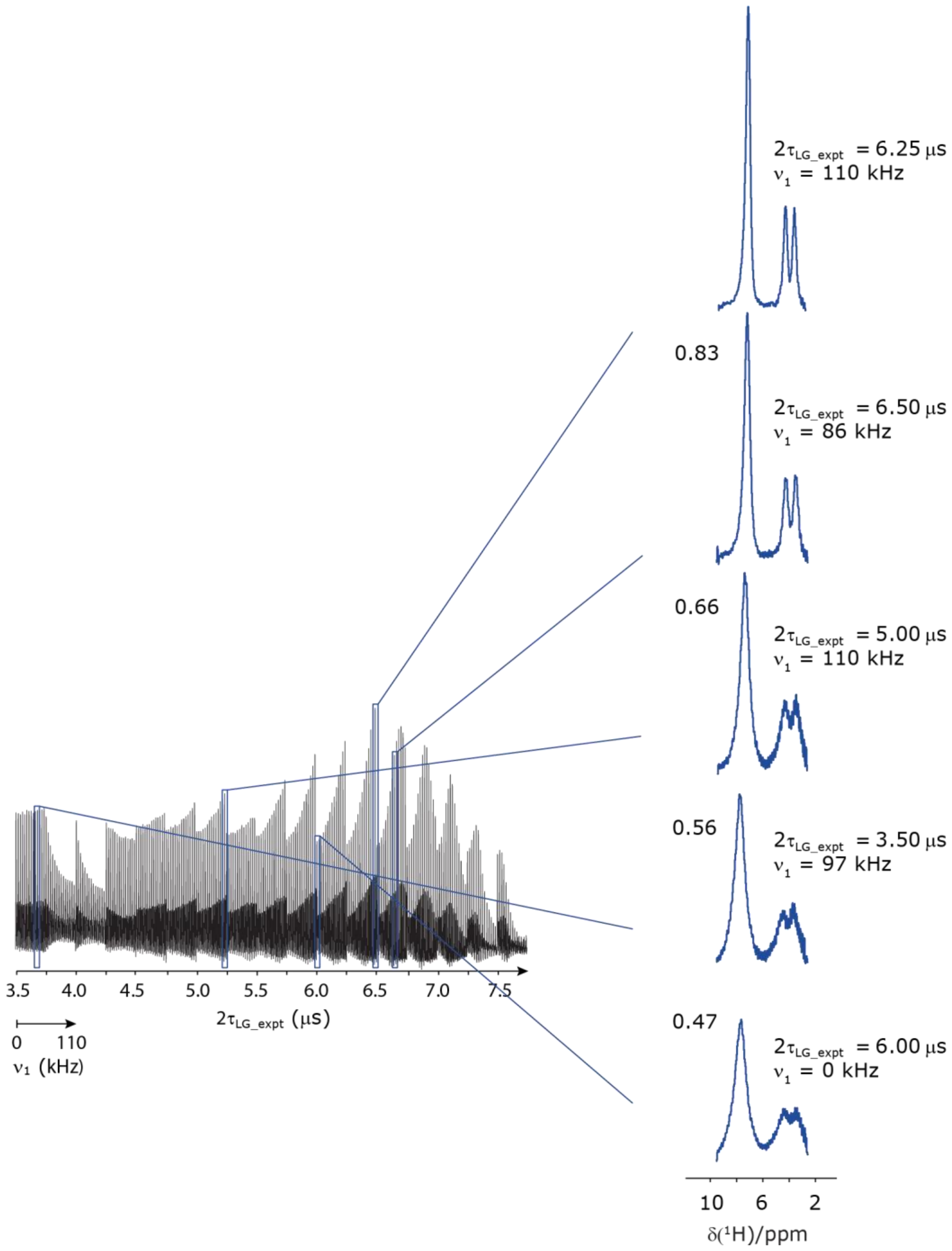

Figure S1. A stacked representation of a two-variable optimization (see **Fig. 3a**) of both τ_{LG_expt} (in steps of 0.25 μs) and ν_1 in a 1D ^{1}H-CRAMPS (ν_0 = 500 MHz) MAS (ν_r = 60 kHz) NMR experiment of ^{15}N-glycine, in which windowed $PMLG5_{mm}^{\bar{x}x}$ was applied with τ_{tilt} = 0.54 μs and a ^{1}H transmitter offset of –0.6 kHz, corresponding to the data shown in Figure 3a of the main text. 8 co-added transients were collected for each optimization point. On the right, slices from the optimization are shown with the associated τ_{LG_expt} and ν_1. The relative intensity of the NH_3^+ peak with respect to the best ^{1}H homonuclear decoupling performance at 2 τ_{LG_expt} = 6.25 μs and ν_1 = 110 kHz is stated.

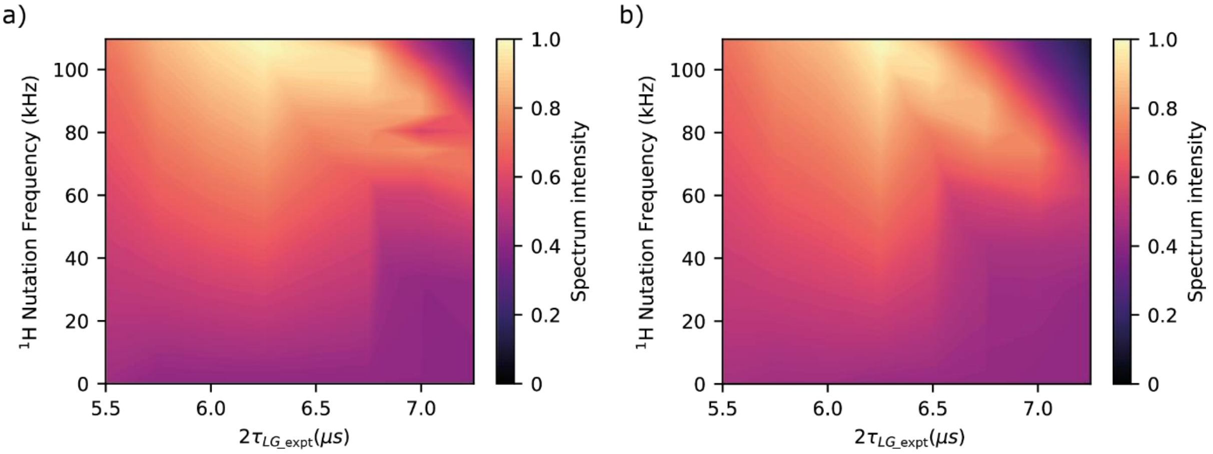

Figure S2. Zoom of the region between τ_{LG_expt} = 5.5 μs – 7.5 μs for the two-variable optimization of τ_{LG_expt} (in steps of 0.25 μs) and ν_1 in a 1D ^{1}H-CRAMPS (ν_0 = 500 MHz) MAS (ν_r = 60 kHz) NMR spectrum of the ^{15}N-glycine a) CH_2 and b) NH_3^+ peak intensity, corresponding to the data shown in Figure 3a of the main text. Windowed $PMLG5_{mm}^{\bar{x}x}$ was applied with τ_w = 7.20 μs, τ_{tilt} = 0.54 μs and a ^{1}H transmitter offset of –0.6 kHz. 8 co-added transients were collected for each optimization point for a recycle delay of 3 s.

S3. Optimisation of tilt pulses via the NH_3^+ signal intensity in a 1D CRAMPS experiment of ^{15}N-glycine

The duration of the tilt pulses, τ_{tilt}, was optimised in a two-variable optimization with τ_{LG_expt}, for the intensity of the NH_3^+ resonance in a 1D CRAMPS spectrum of ^{15}N-glycine at 60 kHz MAS as presented in **Fig. S3a** with windowed $PMLG5_{mm}^{\bar{x}x}$. It is evident from **Fig. S3** that the optimum values for the two parameters, τ_{LG_expt} and τ_{tilt}, are linked, i.e., when one becomes longer the other shortens, maintaining the same combined length of ~7.1 μs (considering two sandwich pulses per $PMLGn_R^{\phi}$ block – see **Fig. 2b**) to maintain the same cycle time, τ_c (see eq. 11), and hence ensure a constant optimum ψ (see eq. 12). The couples with best NH_3^+ signal intensity were 6.75 & 0.15 μs, 6.5 & 0.30 μs and 6.25 & 0.45 μs for 2 τ_{LG_expt} and τ_{tilt}, respectively, with a preference for a longer τ_{LG_expt} and shorter τ_{tilt} (see **Fig. S3b**). A fine optimisation with 16 co-added transients was employed to identify the optimum parameters as used in **Fig. 3c** (and repeated in **Fig. S3c**, left-hand spectrum).

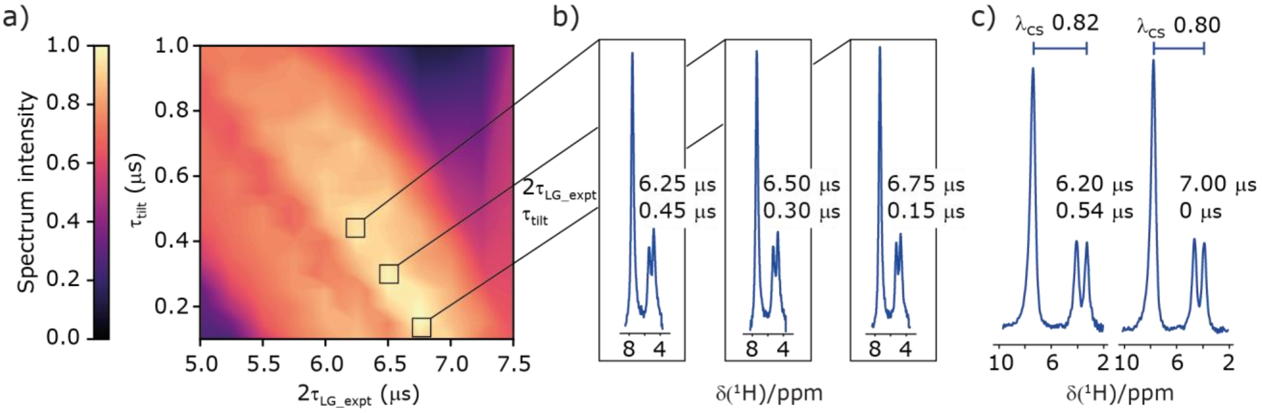

Figure S3. a) Two-variable optimization of 2τ_{LG_expt} (0.25 μs step) and τ_{tilt} (0.05 μs step) for the NH_3^+ peak intensity in a 1D ^{1}H-CRAMPS (ν_0 = 500 MHz) MAS (ν_r = 60 kHz) spectrum of ^{15}N-labelled glycine. Windowed $PMLG5_{mm}^{\bar{x}x}$ was applied with ν_1 = 106 kHz and a ^{1}H transmitter offset of –0.6 kHz. 4 co-added transients were collected for each optimization point. b) Slices extracted from the contour plot show the best spectrum intensities obtained with the indicated 2τ_{LG_expt} and τ_{tilt}. c) 1D ^{1}H CRAMPS ^{15}N-labelled glycine spectra acquired with windowed $PMLG5_{mm}^{\bar{x}x}$ using 2τ_{LG_expt} = 6.20 μs and τ_{tilt} = 0.54 μs (left) and windowed $PMLG5_{mm}^{\bar{x}x}$ without τ_{tilt} (right). 32 co-added transients were added. For all experiments with windowed ^{1}H homonuclear decoupling, τ_w = 7.20 μs.

The ^{1}H CRAMPS spectrum on the right in **Figure S3c** was acquired with the same nutation frequency and offset, but with no tilt pulses and 2τ_{LG_expt} was chosen to be 7 μs such that the cycle time and hence ψ are the same. The intensity of the NH_3^+ peak obtained with windowed $PMLG5_{mm}^{\bar{x}x}$ at τ_{LG_expt} = 6.20 μs and τ_{tilt} = 0.54 μs is within 5% of that obtained without tilt pulses. Note, however, that the peak widths for $PMLG5_{mm}^{\bar{x}x}$ without tilt pulses are 235 Hz for the NH_3^+ peak, and 224 Hz and 231 Hz for the CH_2 peaks. After scaling (λ_{CS} = 0.80), the FWHM become 294 Hz, 280 Hz and 289 Hz, respectively, which is ~15 Hz larger than those stated in **Table 3** for windowed $PMLG5_{mm}^{\bar{x}x}$ with τ_{LG_expt} = 6.20 μs and τ_{tilt} = 0.54 μs.

S4. 2D ^{1}H-^{1}H correlation and optimisation of the ^{15}N-glycine NH_3^+ signal intensity in a 1D-filtered CP-refocused INEPT NMR spectrum for PMLG ^{1}H decoupling

Each ^{1}H-detected FID was acquired for 30 ms with a spectral width of 57 ppm. The ^{1}H indirect dimension was acquired with 96 t_1 FIDs with a dwell time of 29.16 µs (57 ppm spectral width - no ^{1}H homonuclear decoupling), 12.40 µs (134 ppm spectral width - windowless $PMLG5_{mm}^{\bar{x}x}$) and 11.68 µs (143 ppm – windowless $PMLG9_{mm}^{\bar{x}x}$). The maximum t_1 were 1.40 ms, 0.59 ms and 0.56 ms using no ^{1}H homonuclear decoupling, windowless $PMLG5_{mm}^{\bar{x}x}$ and windowless $PMLG9_{mm}^{\bar{x}x}$, respectively. The States-TPPI method was employed to achieve sign discrimination in the indirect dimension.

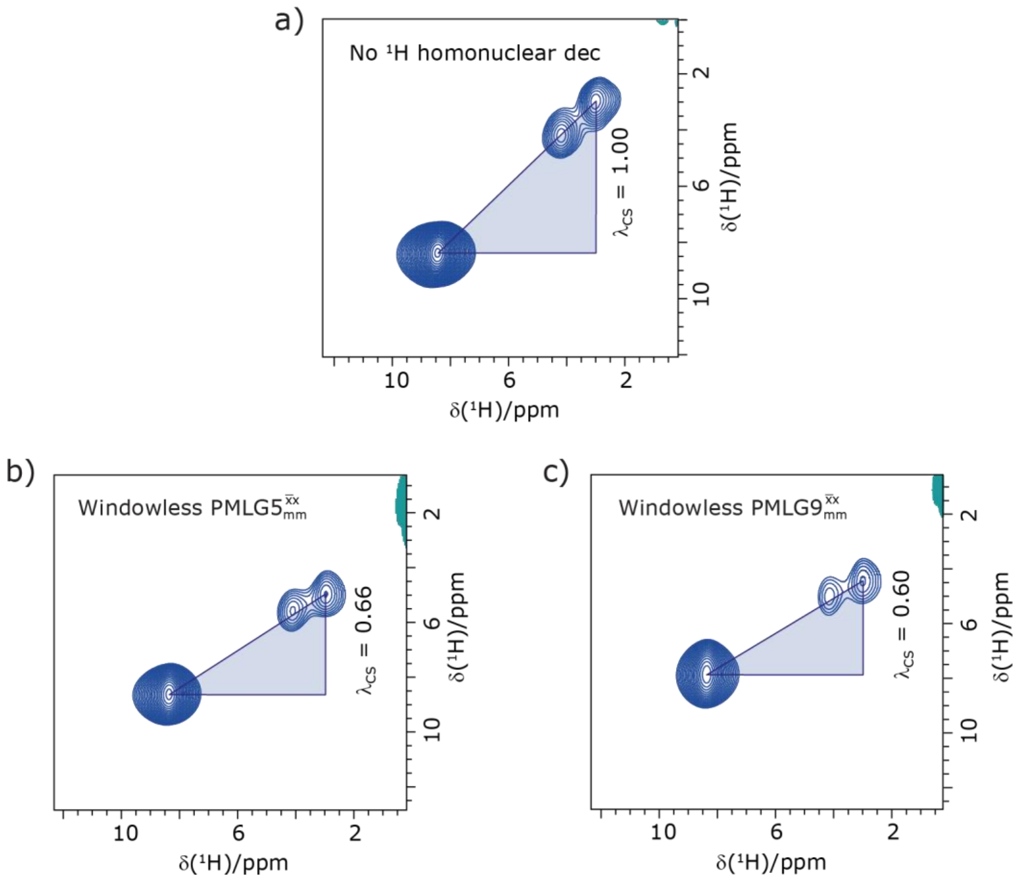

Figure S4. 2D ^{1}H-^{1}H (ν_0 = 600 MHz) correlation spectra of ^{15}N-Glycine acquired at ν_r = 60 kHz MAS with a) no ^{1}H homonuclear decoupling, b) windowless $PMLG5_{mm}^{\bar{x}x}$ (τ_{LG} = 3.10 µs, ν_1 = 104 kHz, Ω = 1 kHz) and c) b) windowless $PMLG9_{mm}^{\bar{x}x}$ (τ_{LG} = 2.92 µs, ν_1 = 104 kHz, Ω = –0.8 kHz). In all the experiments, 4 transients were coadded for 96 t_1 FIDs for a recycle delay of 3 s. The zero-offset is set with the carrier being on resonance with the NH_3^+ peak in the indirect dimension.

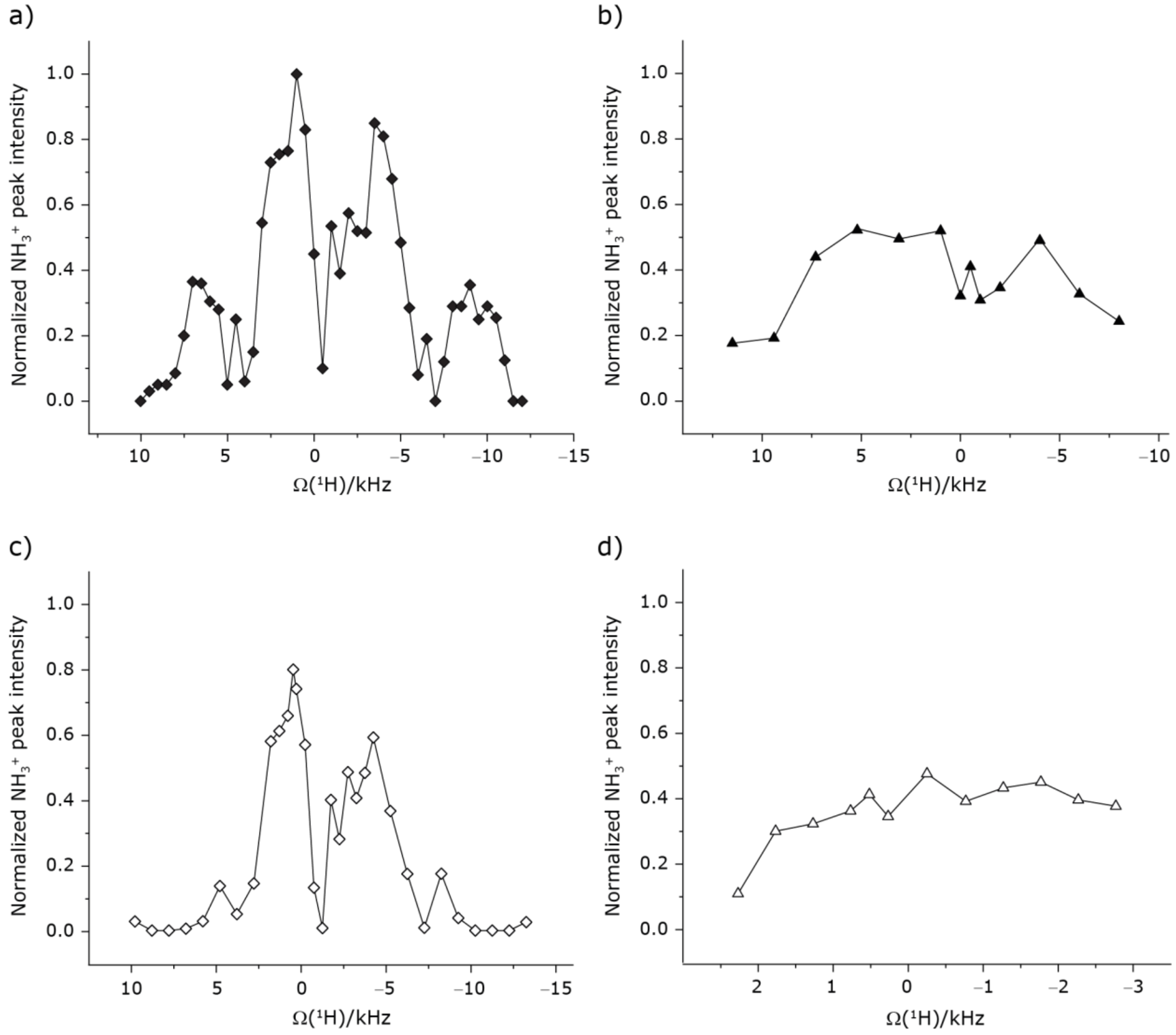

Figure S5. ^{1}H RF carrier optimization for a 1D-filtered (t_1 = 0) ^{15}N-^{1}H (ν_0 = 500 MHz) CP (contact time = 2 ms)-Refocused INEPT MAS (ν_r = 60 kHz) NMR experiment for ^{15}N-labelled glycine, whereby a) windowed $PMLG5_{mm}^{\bar{x}x}$ ^{1}H homonuclear decoupling (See **Fig. 5**) was applied with τ_{LG_expt} = 3.1 µs, τ_{tilt} = 0.54 µs and a ^{1}H nutation frequency, ν_1, of 106 kHz during τ_1 (1.999 ms, 69 τ_c) and 104 kHz during τ_2 (1.391 ms ,48 τ_c), b) windowless $PMLG5_{mm}^{\bar{x}x}$ ^{1}H homonuclear decoupling was applied with τ_{LG_expt} = 3.1 µs and a ^{1}H nutation frequency, ν_1, of 104 kHz during τ_1 (2.096 ms, 169 τ_c) and 102 kHz during τ_2 (0.496 ms, 40 τ_c), c) windowed $PMLG9_{mm}^{\bar{x}x}$ ^{1}H homonuclear decoupling was applied with τ_{LG_expt} = 2.92 µs, τ_{tilt} = 0.82 µs and a ^{1}H nutation frequency, ν_1, of 104 kHz during τ_1 (2.085 ms, 71 τ_c) and 106 kHz during τ_2 (1.498 ms, 51 τ_c) and d) windowless $PMLG9_{mm}^{\bar{x}x}$ ^{1}H homonuclear decoupling was applied with τ_{LG_expt} = 2.92 µs and a ^{1}H nutation frequency, ν_1, of 104 kHz during τ_1 (2.091 ms, 179 τ_c) and 102 kHz during τ_2 (1.192 ms, 102 τ_c). 16 transients were coadded. For all experiments with windowed decoupling, τ_w was substituted with a delay of 7.20 µs. The zero-offset is set with the carrier being on resonance with the NH_3^+ peak.

S5. Cimetidine

Here, the normalized intensity is related to the respective maximum intensity for each peak , i.e. the maximum intensity is equal to 1 for all the resonances. However, note that the NH15 proton signal intensity is ~30 % of that of NH3.

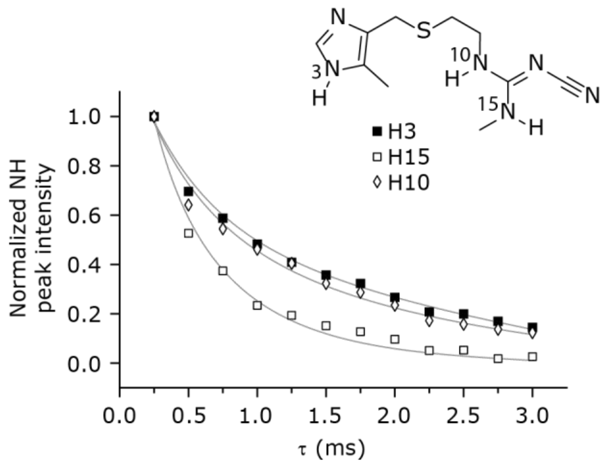

Figure S6. Dephasing of cimetidine NH proton (ν_0 = 600 MHz) resonances as a function of the spin-echo duration, τ, with windowed $PMLG5^{\bar{x}x}_{mm}$ (τ_{LG_expt} = 3.10 µs, τ_{tilt} = 0.54 µs and τ_w = 7.20 µs) for a nutation frequency of 106 kHz. Fits to an exponential decay function are shown, with the spin-echo dephasing times, T_2', as listed in **Table S1**. 8 transients were co-added for a recycle delay of 5 s.

Table S1. Cimetidine ^{1}H dephasing time, T_2', for the three NH resonances and T_2' scaled by the experimental λ_{CS},, λ_{CS} T_2', acquired on a ^{1}H spin-echo[a] experiment using windowed $PMLG5^{\bar{x}x}_{mm}$ [b]

	δ (ppm)	ν_1 (kHz)	λ_{CS}	T_2' (ms)	λ_{CS} T_2' (ms)
NH3	11.6	106	0.82	1.34	1.10
NH15	9.7			0.58	0.48
NH10	8.2			1.23	1.01

[a]Implemented at ν_0 = 600 MHz and ν_r = 60 kHz (see Fig. S6). Windowed $PMLG5^{\bar{x}x}_{mm}$ was implemented with τ_{LG} = 3.10 µs, τ_{tilt} = 0.54 µs and τ_w = 7.20 µs

[b] Ω_{rf} = – 0.8 kHz, where the zero-offset is set with the carrier being on resonance with the NH_3^+ peak of ^{15}N-glycine

S6. Simulations of eqs 1 and 2

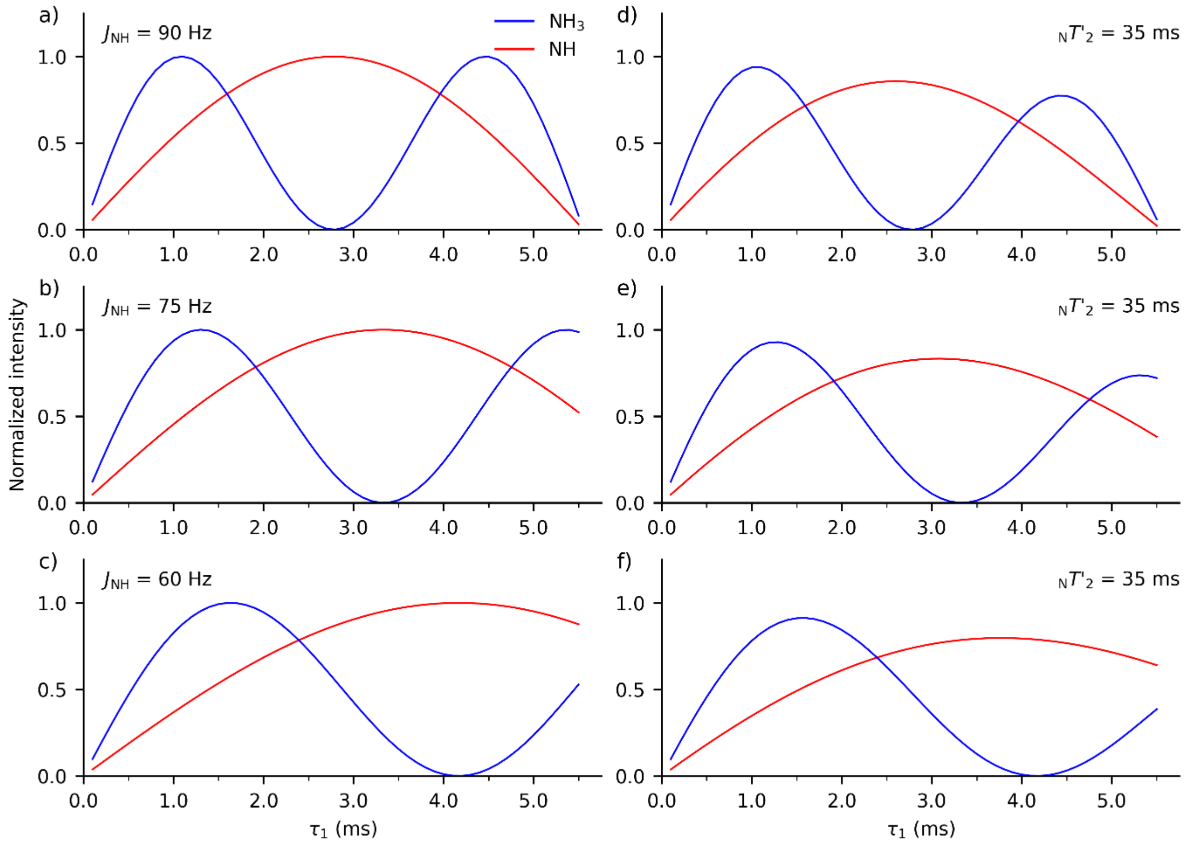

Figure S7. Simulation of dependence of the ^{15}N-^{1}H CP-Refocused INEPT intensity on the spin-echo period, τ_1, according to eq. 1 and 2 (from the main text) for a NH (red) or NH_3 (blue) group, for a J_{NH} equal to: a) 90 Hz, b) 75 Hz and c) 60 Hz ignoring dephasing, and d) 90 Hz, e) 75 Hz and f) 60 Hz with exponential dephasing with a nominal nitrogen T_2' of 35 ms.
